# Supplementary material for: Circular RNA circLDLR facilitates cancer progression by altering the miR-30a-3p/SOAT1 axis in colorectal cancer
Source: Cell Death Discov. 2022 Jul 11;8:314. doi: 10.1038/s41420-022-01110-5 (PMC9276972; doi:10.1038/s41420-022-01110-5)
Supplement: Supplementary file 1 — Supplementary Table S1 [file 41420_2022_1110_MOESM1_ESM.docx]

**Supplementary Table S1 Detailed** **clinicopathological characteristics of the five paired tissues.**

| **Number** | **Gender** | **Age** | **Position** | **Stages** |
| --- | --- | --- | --- | --- |
| 1 | Male | 62 | Rectum | ⅡA |
| 2 | Male | 51 | Colon | ⅡA |
| 3 | Female | 58 | Colon | ⅢB |
| 4 | Male | 59 | Colon | ⅢB |
| 5 | Male | 57 | Colon | ⅢB |
