# Supplementary material for: Circular RNA circLDLR facilitates cancer progression by altering the miR-30a-3p/SOAT1 axis in colorectal cancer
Source: Cell Death Discov. 2022 Jul 11;8:314. doi: 10.1038/s41420-022-01110-5 (PMC9276972; doi:10.1038/s41420-022-01110-5)
Supplement: Supplementary file 2 — Supplementary Table S2 [file 41420_2022_1110_MOESM2_ESM.docx]

**Supplementary Table S2 Primers and DNA/RNA sequences used in this study**

| Item | Primer Sequence(5’-3’） |
| --- | --- |
| Primers |  |
| hsa-GAPDH-Forward | GGAGCGAGATCCCTCCAAAAT |
| hsa-GAPDH-Reverse | GGCTGTTGTCATACTTCTCATGG |
| hsa-circLDLR-Forward | GAGCTGCCTCACAGGACAAAG |
| hsa-circLDLR-Reverse | TGAGGTTGTGGAAGAGAACCATATC |
| hsa-LDLR-Forward | TCTGCAACATGGCTAGAGACT |
| hsa-LDLR-Reverse | TCCAAGCATTCGTTGGTCCC |
| hsa-miR-342-3p-Forward | ACACTCCAGCTGGGTCTCACACAGAAATCGC |
| hsa-miR-342-3p-Reverse | CTCAACTGGTGTCGTGGAGTCGGCAATTCAGTTGAGACGGGTGC |
| hsa-miR-30e-3p-Forward | ACACTCCAGCTGGGCTTTCAGTCGGATGTT |
| hsa-miR-30e-3p-Reverse | CTCAACTGGTGTCGTGGAGTCGGCAATTCAGTTGAGAAGACGGG |
| hsa-miR-483-3p-Forward | ACACTCCAGCTGGGTCACTCCTCTCCTCC |
| hsa-miR-483-3p-Reverse | CTCAACTGGTGTCGTGGAGTCGGCAATTCAGTTGAGAAGACGGG |
| hsa-miR-15b-5p-Forward | ACACTCCAGCTGGGTAGCAGCACATCATGG |
| hsa-miR-15b-5p-Reverse  hsa-miR-744-5p-Forward | CTCAACTGGTGTCGTGGAGTCGGCAATTCAGTTGAGTGTAAACC  ACACTCCAGCTGGGTGCGGGGCTAGGGCTA |
| hsa-miR-744-5p-Reverse | CTCAACTGGTGTCGTGGAGTCGGCAATTCAGTTGAGTGCTGTTA |
| hsa-miR-326-Forward | ACACTCCAGCTGGGCCTCTGGGCCCTTC |
| hsa-miR-326-Reverse | CTCAACTGGTGTCGTGGAGTCGGCAATTCAGTTGAGCTGGAGGA |
| hsa-miR-15a-5p-Forward | ACACTCCAGCTGGGTAGCAGCACATAATGG |
| hsa-miR-15a-5p-Reverse | CTCAACTGGTGTCGTGGAGTCGGCAATTCAGTTGAGCACAAACC |
| hsa-miR-30d-3p-Forward | ACACTCCAGCTGGGCTTTCAGTCAGATGTT |
| hsa-miR-30d-3p-Reverse | CTCAACTGGTGTCGTGGAGTCGGCAATTCAGTTGAGGCAGCAAA |
| hsa-miR-30a-3p-Forward | ACACTCCAGCTGGGCTTTCAGTCGGATGTT |
| hsa-miR-30a-3p-Reverse | CTCAACTGGTGTCGTGGAGTCGGCAATTCAGTTGAGGCTGCAAA |
| hsa-miR-16-5p-Forward | ACACTCCAGCTGGGTAGCAGCACGTAAATA |
| hsa-miR-16-5p-Reverse | CTCAACTGGTGTCGTGGAGTCGGCAATTCAGTTGAGCGCCAATA |
| hsa-miR-146a-5p-Forward | ACACTCCAGCTGGGTGAGAACTGAATTCCA |
| hsa-miR-146a-5p-Reverse | CTCAACTGGTGTCGTGGAGTCGGCAATTCAGTTGAGAACCCATG |
| hsa-URP | TGGTGTCGTGGAGTCG |
| hsa-U6-Forward | CTCGCTTCGGCAGCACA |
| hsa-U6-Reverse | AACGCTTCACGAATTTGCGT |
| hsa-SOAT1-Forward | GAAGTTGGCAGTCACTTTGATGA |
| hsa-SOAT1-Reverse | GAGCGCACCCACCATTATCTA |
| hsa-HMGCR-Forward | TGATTGACCTTTCCAGAGCAAG |
| hsa-HMGCR-Reverse | CTAAAATTGCCATTCCACGAGC |
| hsa-EIF4A3-Forward | GGGGCATCTACGCTTACGG |
| hsa-EIF4A3-Reverse | GCGATGACATCTCTCCCTTTGA |
| hsa-METTL3-Forward | TTGTCTCCAACCTTCCGTAGT |
| hsa-METTL3-Reverse | CCAGATCAGAGAGGTGGTGTAG |
| hsa-METTL14-Forward | GAACACAGAGCTTAAATCCCCA |
| hsa-METTL14-Reverse | TGTCAGCTAAACCTACATCCCTG |
| hsa-WTAP-Forward | CCTCCCTTCACCTTTCCTC |
| hsa-WTAP-Reverse | TGGGAAGAGGTTCTTCGTTG |
| miRNA mimics and inhibitors |  |
| miR-30a-3p mimics | CUUUCAGUCGGAUGUUUGCAGC+UGCAAACAUCCGACUGAAAGUU |
| miR-30a-3p inhibitors | GCUGCAAACAUCCGACUGAAAG |
| Probes |  |
| hsa-circLDLR FISH probe | AATACTTTGTCCTGTGAGGCAGC |
| hsa-circLDLR pulldown probe | UCUGUCCAAAA+UACUUUGUCCUG+UCCUCAUG |
| miR-30a-3p FISH probe | GCTGCAAACA+TCCGACTGAAAG |
| siRNAs |  |
| si-circLDLR-1 | CTGCCTCACAGGACAAAGT |
| si-circLDLR-2 | TGCCTCACAGGACAAAGTA |
| si-circLDLR-3 | GCCTCACAGGACAAAGTAT |
| si-circLDLR-4 | GAGCUGCCUCACAGGACAATT+UUGUCCUGUGAGGCAGCUCTT |
| si-circLDLR-5 | UCACAGGACAAAGUAUUUUTT+AAAAUACUUUGUCCUGUGATT |
| si-EIF4A3-1 | CGAGCAATCAAGCAGATCA |
| si-EIF4A3-2 | GCTGGATTACGGACAGCAT |
| si-EIF4A3-3 | CTCTCGGTGACTACATGAA |
