# Supplementary material for: Circular RNA circLDLR facilitates cancer progression by altering the miR-30a-3p/SOAT1 axis in colorectal cancer
Source: Cell Death Discov. 2022 Jul 11;8:314. doi: 10.1038/s41420-022-01110-5 (PMC9276972; doi:10.1038/s41420-022-01110-5)
Supplement: Supplementary file 3 — Supplementary Table S3 [file 41420_2022_1110_MOESM3_ESM.docx]

**Supplementary Table S3 Correlations between the expression of circLDLR and clinicalpathological features in 75 CRC patients**

| Characteristic | Cases | CircLDLR expression | | P-value |
| --- | --- | --- | --- | --- |
|  |  | Low | High |  |
| All cases | 75 |  |  |  |
| Gender |  |  |  |  |
| Male | 43 | 28 | 15 | 0.535 |
| Female | 32 | 23 | 9 |  |
| Age (years) |  |  |  |  |
| <70 | 45 | 33 | 12 | 0.225 |
| ≥70 | 30 | 18 | 12 |  |
| T stage |  |  |  |  |
| T1-T3 | 21 | 16 | 5 | 0.343 |
| T4 | 54 | 35 | 19 |  |
| N stage |  |  |  |  |
| N0/N1 | 64 | 47 | 17 | 0.015* |
| N2/N3 | 11 | 4 | 7 |  |
| Tumor grade |  |  |  |  |
| Low | 57 | 39 | 18 | 0.889 |
| High | 18 | 12 | 6 |  |
