# Supplementary material for: Circular RNA circLDLR facilitates cancer progression by altering the miR-30a-3p/SOAT1 axis in colorectal cancer
Source: Cell Death Discov. 2022 Jul 11;8:314. doi: 10.1038/s41420-022-01110-5 (PMC9276972; doi:10.1038/s41420-022-01110-5)
Supplement: Supplementary file 4 — Supplementary Table S4 [file 41420_2022_1110_MOESM4_ESM.docx]

**Supplementary Table S4 109 candidate miRNAs potentially interacted with circLDLR**

| Number | miRNA | Number | miRNA | Number | miRNA |
| --- | --- | --- | --- | --- | --- |
| 1 | hsa-miR-6834-3p | 38 | hsa-miR-8089 | 75 | hsa-miR-3934-3p |
| 2 | hsa-miR-6833-5p | 39 | hsa-miR-1827 | 76 | hsa-miR-6775-5p |
| 3 | hsa-miR-4710 | 40 | hsa-miR-330-5p | 77 | hsa-miR-6893-5p |
| 4 | hsa-miR-6736-3p | 41 | hsa-miR-103a-3p | 78 | hsa-miR-6721-5p |
| 5 | hsa-miR-16-5p | 42 | hsa-miR-483-3p | 79 | hsa-miR-107 |
| 6 | hsa-miR-3665 | 43 | hsa-miR-6751-5p | 80 | hsa-miR-5090 |
| 7 | hsa-miR-6881-3p | 44 | hsa-miR-650 | 81 | hsa-miR-4649-5p |
| 8 | hsa-miR-4706 | 45 | hsa-miR-92a-1-5p | 82 | hsa-miR-4745-5p |
| 9 | hsa-miR-497-5p | 46 | hsa-miR-15b-5p | 83 | hsa-miR-1228-5p |
| 10 | hsa-miR-12128 | 47 | hsa-miR-744-5p | 84 | hsa-miR-1321 |
| 11 | hsa-miR-146a-5p | 48 | hsa-miR-326 | 85 | hsa-miR-9903 |
| 12 | hsa-miR-589-5p | 49 | hsa-miR-3157-5p | 86 | hsa-miR-7111-3p |
| 13 | hsa-miR-1266-5p | 50 | hsa-miR-1296-3p | 87 | hsa-miR-30d-3p |
| 14 | hsa-miR-6838-5p | 51 | hsa-miR-6729-5p | 88 | hsa-miR-4660 |
| 15 | hsa-miR-4736 | 52 | hsa-miR-4700-5p | 89 | hsa-miR-3682-3p |
| 16 | hsa-miR-4734 | 53 | hsa-miR-6776-5p | 90 | hsa-miR-6766-5p |
| 17 | hsa-miR-4739 | 54 | hsa-miR-4316 | 91 | hsa-miR-4268 |
| 18 | hsa-miR-4667-5p | 55 | hsa-miR-12125 | 92 | hsa-miR-5787 |
| 19 | hsa-miR-424-5p | 56 | hsa-miR-6086 | 93 | hsa-miR-96-5p |
| 20 | hsa-miR-4518 | 57 | hsa-miR-6754-5p | 94 | hsa-miR-1207-5p |
| 21 | hsa-miR-342-3p | 58 | hsa-miR-15a-5p | 95 | hsa-miR-6803-5p |
| 22 | hsa-miR-6852-3p | 59 | hsa-miR-6756-5p | 96 | hsa-miR-9986 |
| 23 | hsa-miR-30e-3p | 60 | hsa-miR-4435 | 97 | hsa-miR-3909 |
| 24 | hsa-miR-5589-5p | 61 | hsa-miR-377-5p | 98 | hsa-miR-4270 |
| 25 | hsa-miR-4472 | 62 | hsa-miR-486-3p | 99 | hsa-miR-3925-5p |
| 26 | hsa-miR-6774-5p | 63 | hsa-miR-4645-3p | 100 | hsa-miR-6736-5p |
| 27 | hsa-miR-3622a-5p | 64 | hsa-miR-7112-5p | 101 | hsa-miR-3612 |
| 28 | hsa-miR-6077 | 65 | hsa-miR-6868-5p | 102 | hsa-miR-1293 |
| 29 | hsa-miR-4505 | 66 | hsa-miR-4441 | 103 | hsa-miR-1294 |
| 30 | hsa-miR-4447 | 67 | hsa-miR-4763-3p | 104 | hsa-miR-2355-3p |
| 31 | hsa-miR-6780a-3p | 68 | hsa-miR-6808-5p | 105 | hsa-miR-6765-5p |
| 32 | hsa-miR-4756-5p | 69 | hsa-miR-3191-3p | 106 | hsa-miR-30a-3p |
| 33 | hsa-miR-323a-5p | 70 | hsa-miR-6810-5p | 107 | hsa-miR-4481 |
| 34 | hsa-miR-4701-3p | 71 | hsa-miR-4530 | 108 | hsa-miR-10396a-5p |
| 35 | hsa-miR-6842-3p | 72 | hsa-miR-6746-3p | 109 | hsa-miR-3190-5p |
| 36 | hsa-miR-3197 | 73 | hsa-miR-1262 |  |  |
| 37 | hsa-miR-3190-3p | 74 | hsa-miR-4483 |  |  |
