# Supplementary material for: Circular RNA circLDLR facilitates cancer progression by altering the miR-30a-3p/SOAT1 axis in colorectal cancer
Source: Cell Death Discov. 2022 Jul 11;8:314. doi: 10.1038/s41420-022-01110-5 (PMC9276972; doi:10.1038/s41420-022-01110-5)
Supplement: Supplementary file 5 — Supplementary Table S5 [file 41420_2022_1110_MOESM5_ESM.docx]

**Supplementary Table S****5 11 potential target miRNAs were selected for further analysis**

| Number | miRNA |
| --- | --- |
| 1 | hsa-miR-342-3p |
| 2 | hsa-miR-30e-3p |
| 3 | hsa-miR-483-3p |
| 4 | hsa-miR-15b-5p |
| 5 | hsa-miR-744-5p |
| 6 | hsa-miR-326 |
| 7 | hsa-miR-15a-5p |
| 8 | hsa-miR-30d-3p |
| 9 | hsa-miR-30a-3p |
| 10 | hsa-miR-16-5p |
| 11 | hsa-miR-146a-5p |
