# Supplementary material for: Circular RNA circLDLR facilitates cancer progression by altering the miR-30a-3p/SOAT1 axis in colorectal cancer
Source: Cell Death Discov. 2022 Jul 11;8:314. doi: 10.1038/s41420-022-01110-5 (PMC9276972; doi:10.1038/s41420-022-01110-5)
Supplement: Supplementary file 6 — Supplementary Figure legend [file 41420_2022_1110_MOESM6_ESM.docx]

**Supplementary figure legends**

**Figure S1.** The differentially expressed circRNAs in CRC.

**Figure S2.** The ability of circLDLR to encode protein. The ability of circLDLR to encode protein was analyzed by circRNADb.

**Figure S3.** The stable CRC cells were established. **a** The stable circLDLR knockdown (sh-circLDLR) RKO and HCT116 cells were established and verified by qRT-PCR. **b** The stable circLDLR overexpression (circLDLR-OE) SW480 and HT29 cells were established and verified by qRT-PCR.

**Figure S4.** The expression of the potential target miRNAs in CRC cells.

**Figure S5.** SOAT1 is a downstream target of miR-30a-3p. **a**, **b** The expression of HMGCR in RKO (**a**) and SW480 (**b**) cells. **c** Schematic illustration of SOAT1-WT and SOAT1-Mut luciferase reporter vectors.

**Figure S6.** The circLDLR/miR-30a-3p/SOAT1 axis modulates malignant behaviour and increases cholesterol levels in CRC. **a** EdU analysis of the cell proliferation ability in SW480 and HT29 transfected with miR-30a-3p inhibitors or cotransfected with si-SOAT1 and miR-30a-3p inhibitors. Representative images are shown. Scale bar, 200 μm. **b** Cell migration and invasion in SW480 and HT29 transfected with miR-30a-3p inhibitors or cotransfected with si-SOAT1 and miR-30a-3p inhibitors were examined. Representative images are shown. Scale bar, 200 μm. **c** EdU analysis of the cell proliferation ability in SW480 and HT29 transfected with circLDLR-OE or cotransfected with si-SOAT1 and circLDLR-OE. Representative images are shown. Scale bar, 200 μm. **d** Cell migration and invasion in SW480 and HT29 transfected with circLDLR-OE or cotransfected with si-SOAT1 and circLDLR-OE were examined. Representative images are shown. Scale bar, 200 μm.

**Figure S7.** CircLDLR promotes CRC tumor growth in vivo. **a** Representative IHC staining images of subcutaneous tumors revealed the relative protein levels of Ki-67, CD31 and SOAT1 in sh-circLDLR and sh-NC groups. Scale bar, 100 μm. Statistical analysis of the relative protein levels of Ki-67, CD31 and SOAT1 in in sh-circLDLR and sh-NC groups. **b** Representative IHC staining images of subcutaneous tumors revealed the relative protein levels of Ki-67, CD31 and SOAT1 in circLDLR-OE and Vector groups. Scale bar, 100 μm. Statistical analysis of the relative protein levels of Ki-67, CD31 and SOAT1 in circLDLR-OE and Vector groups.
