# Supplementary figures and images for: Circular RNA circLDLR facilitates cancer progression by altering the miR-30a-3p/SOAT1 axis in colorectal cancer
Source: Cell Death Discov. 2022 Jul 11;8:314. doi: 10.1038/s41420-022-01110-5 (PMC9276972; doi:10.1038/s41420-022-01110-5)

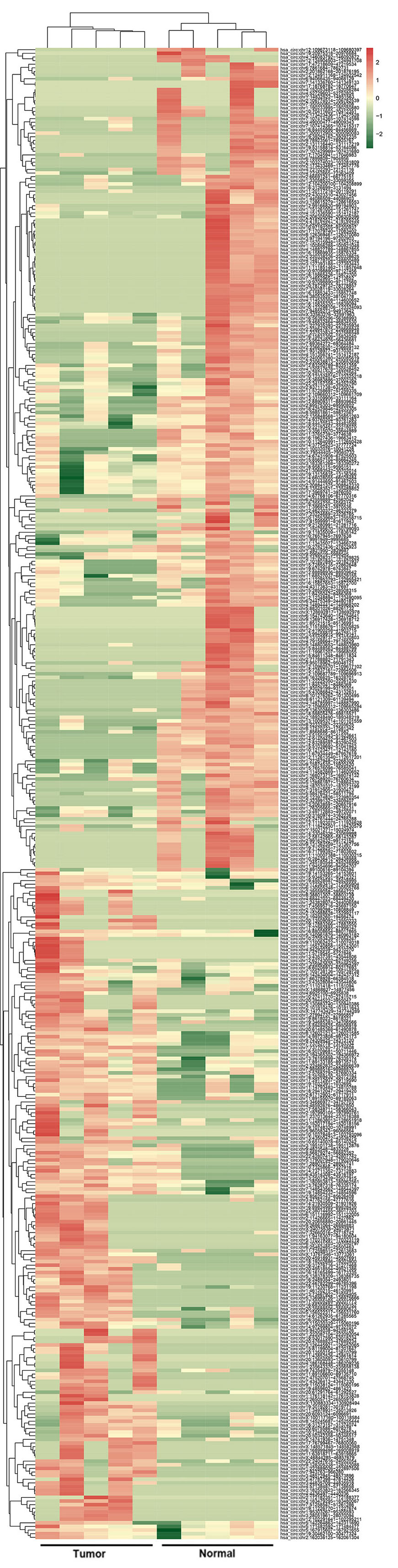

Supplement: Supplementary file 7 — Supplementary Fig. S1 [file 41420_2022_1110_MOESM7_ESM.jpg]

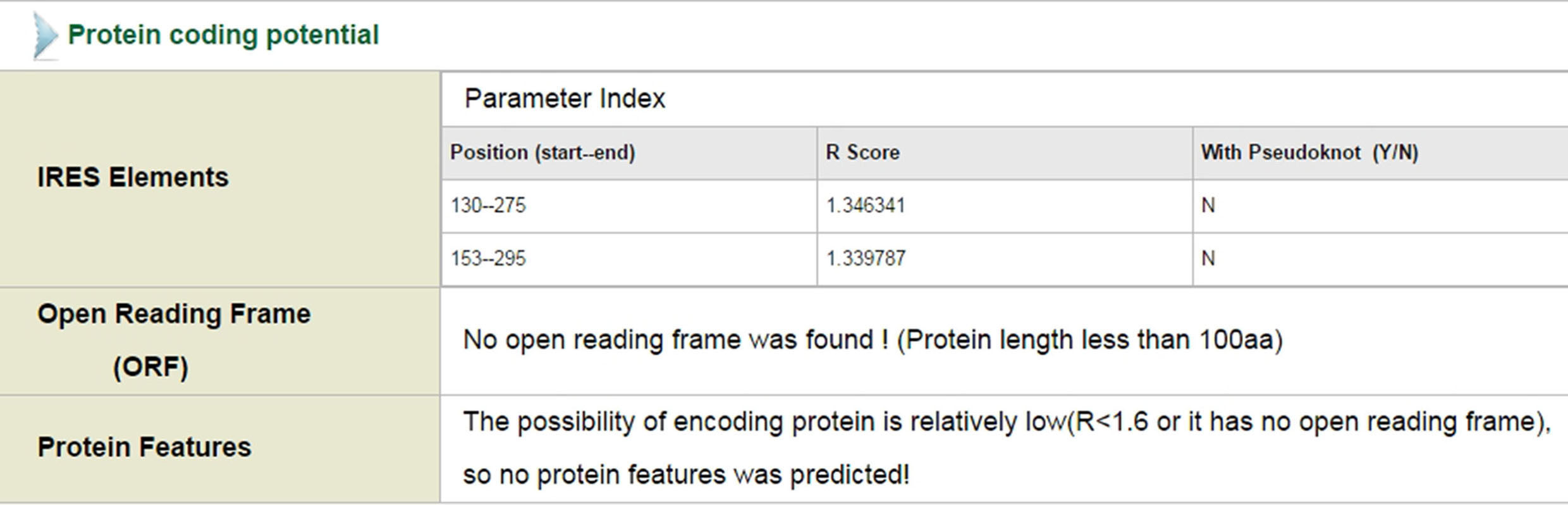

Supplement: Supplementary file 8 — Supplementary Fig. S2 [file 41420_2022_1110_MOESM8_ESM.jpg]

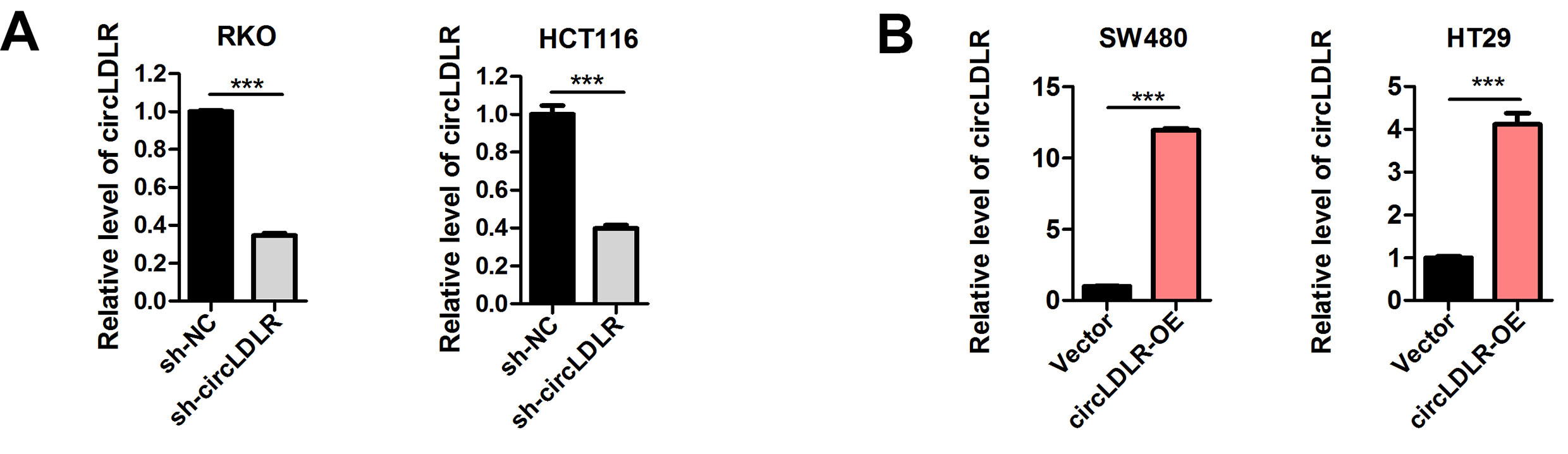

Supplement: Supplementary file 9 — Supplementary Fig. S3 [file 41420_2022_1110_MOESM9_ESM.jpg]

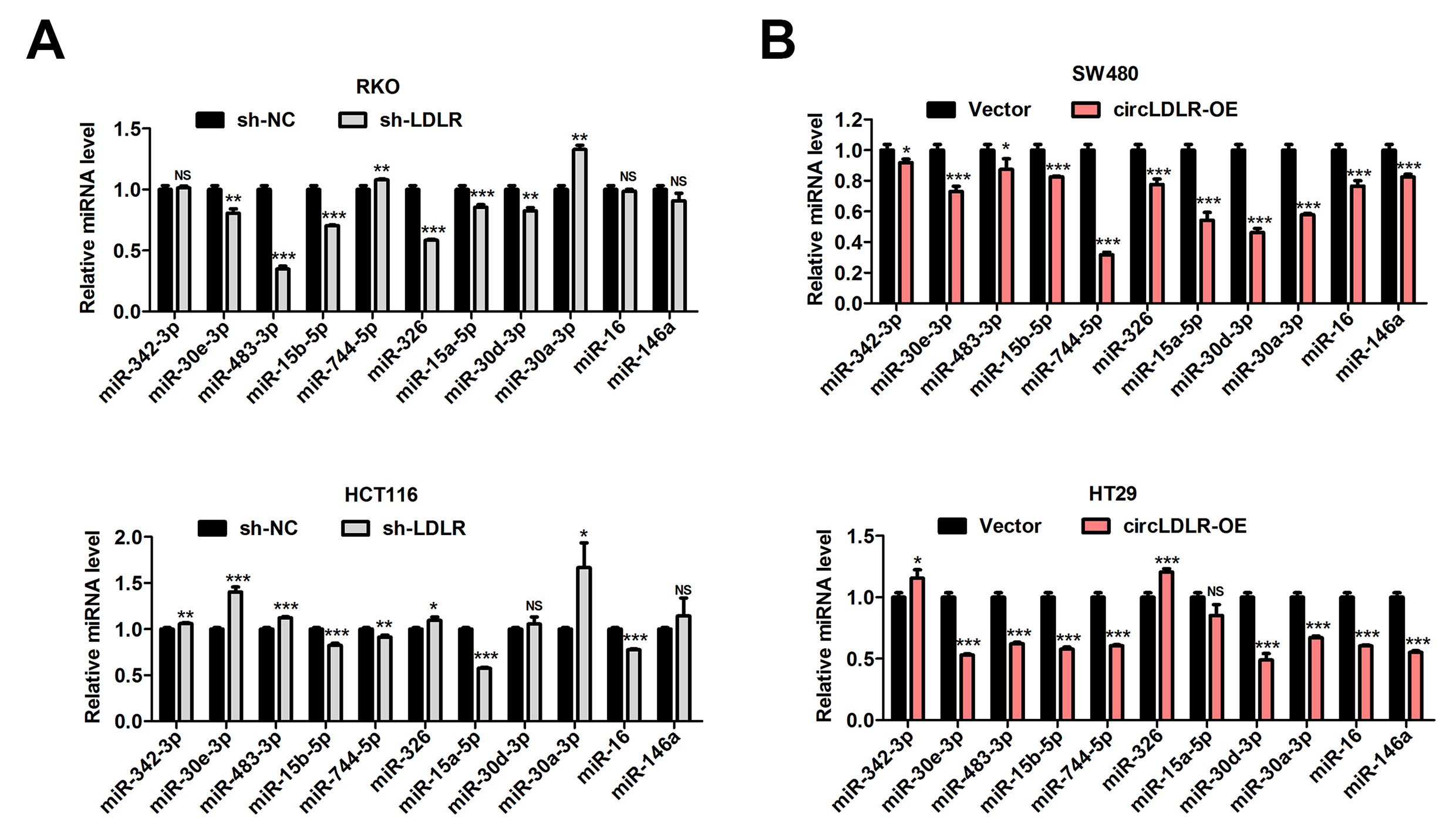

Supplement: Supplementary file 10 — Supplementary Fig. S4 [file 41420_2022_1110_MOESM10_ESM.jpg]

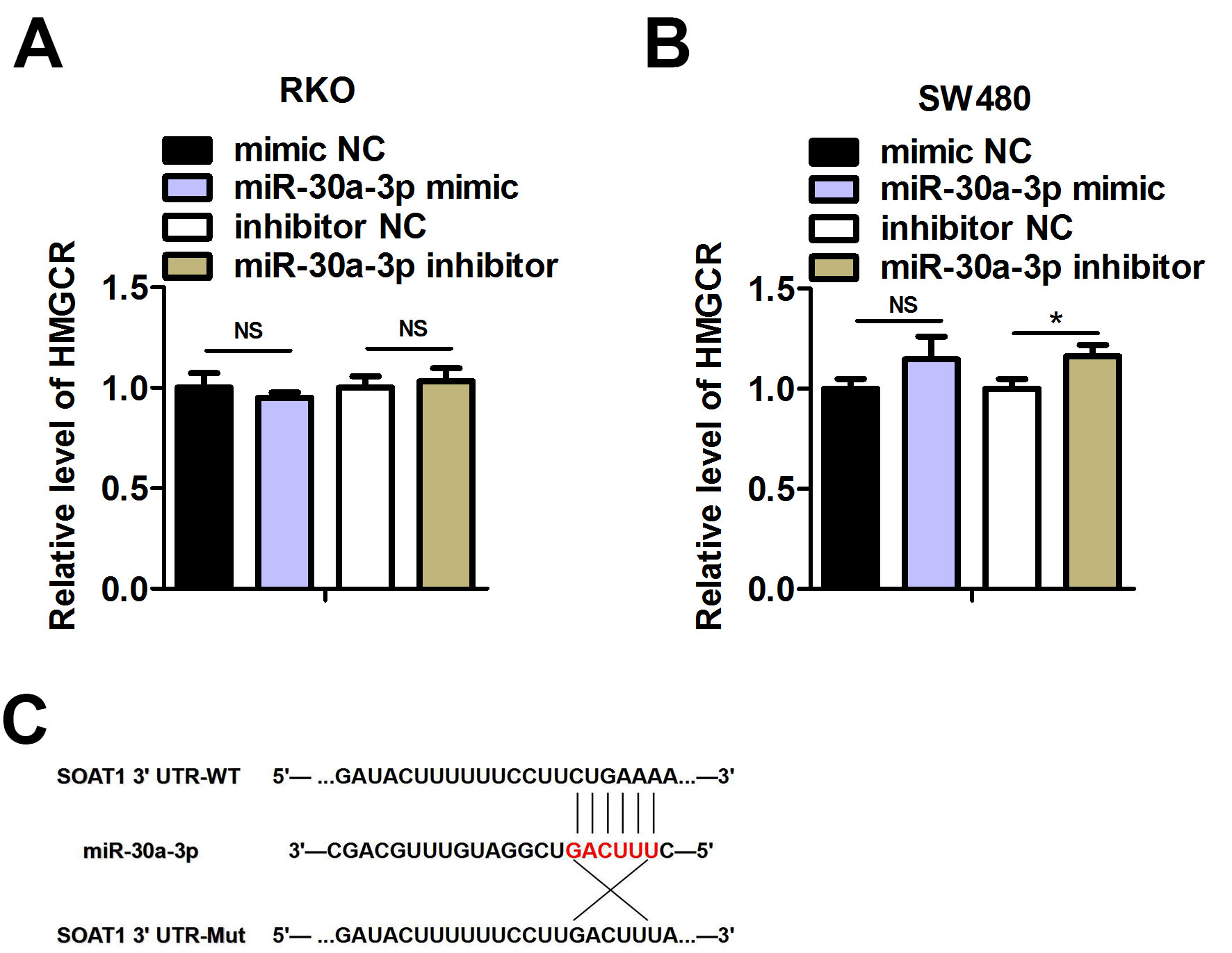

Supplement: Supplementary file 11 — Supplementary Fig. S5 [file 41420_2022_1110_MOESM11_ESM.jpg]

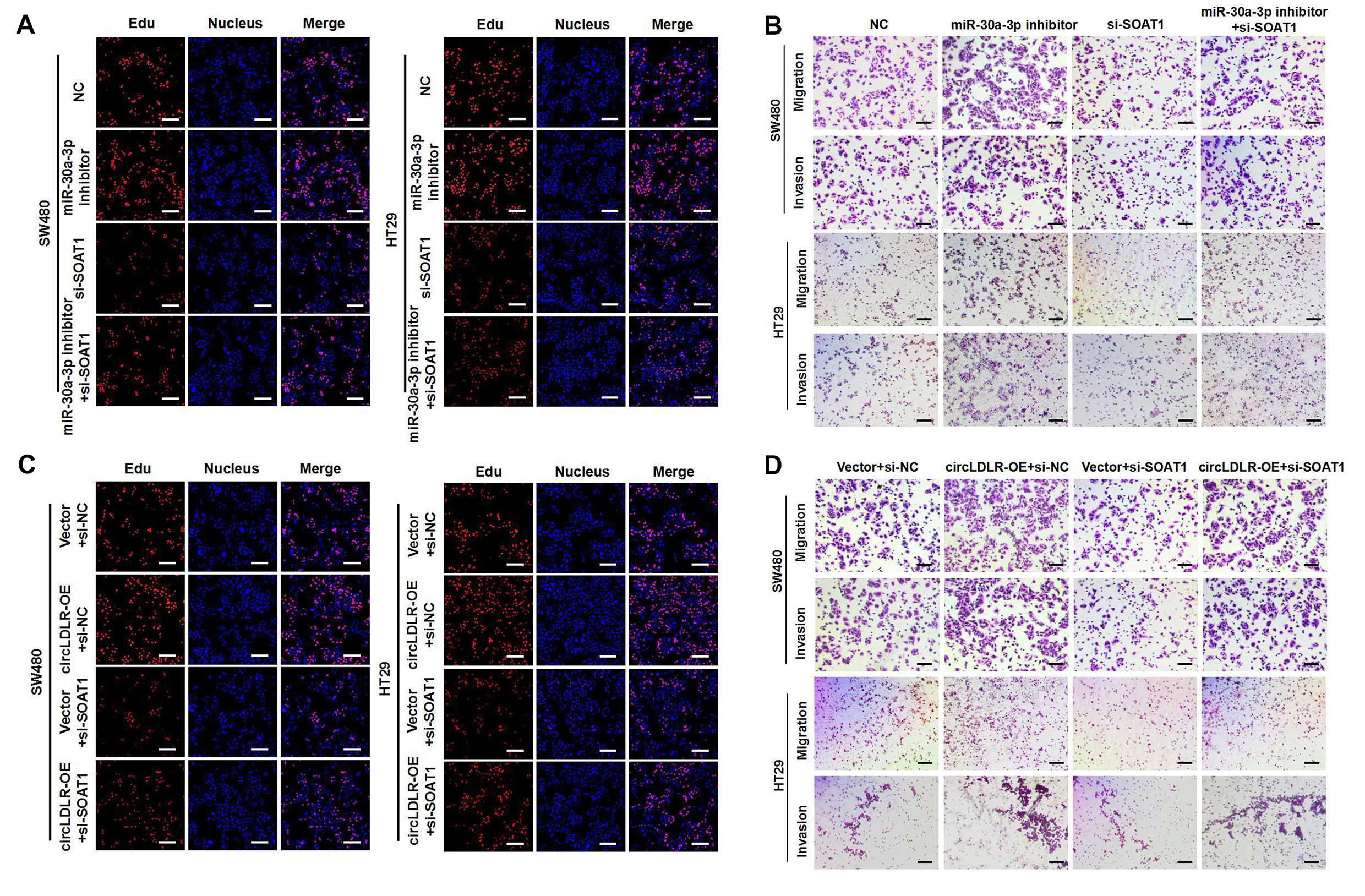

Supplement: Supplementary file 12 — Supplementary Fig. S6 [file 41420_2022_1110_MOESM12_ESM.jpg]

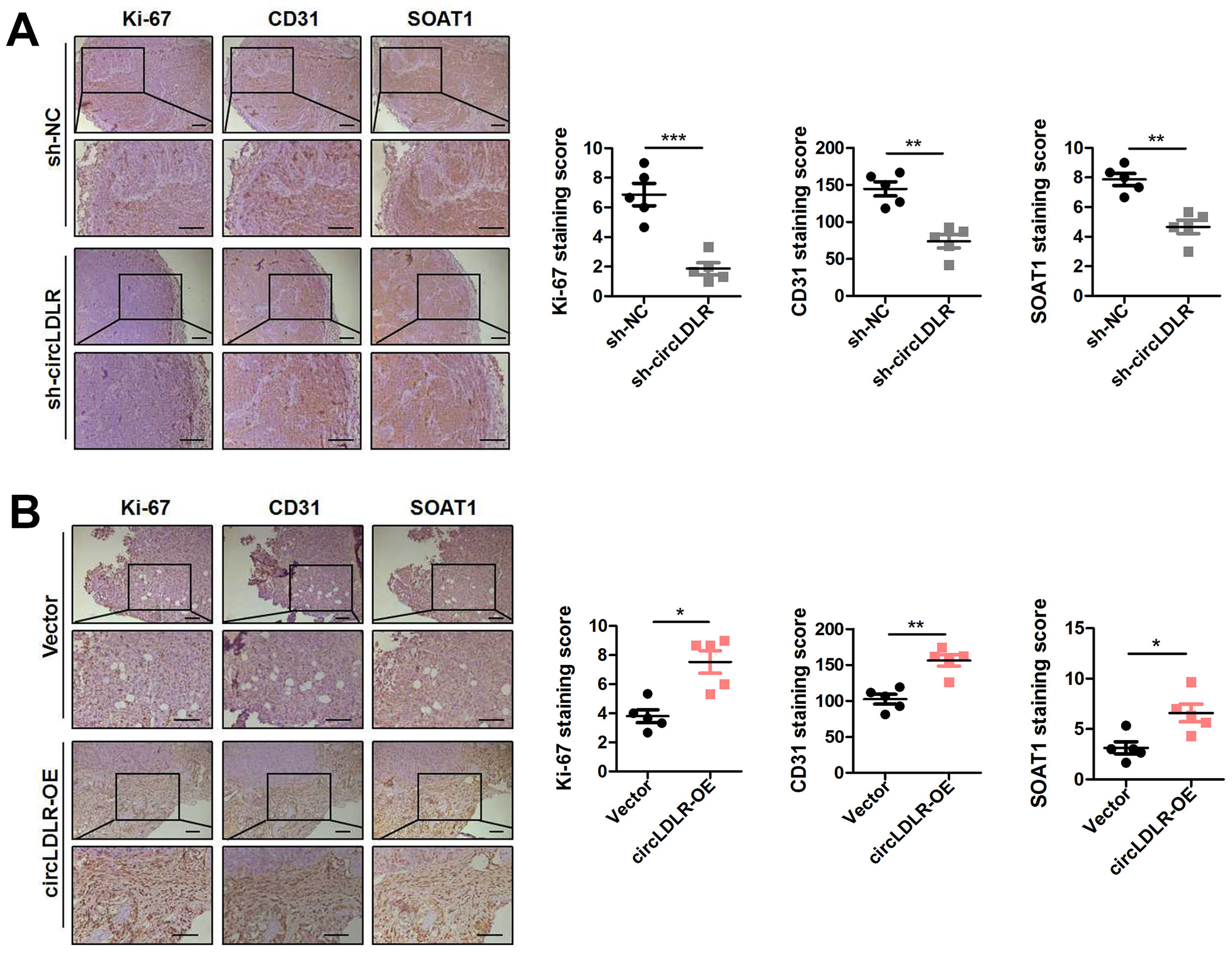

Supplement: Supplementary file 13 — Supplementary Fig. S7 [file 41420_2022_1110_MOESM13_ESM.jpg]

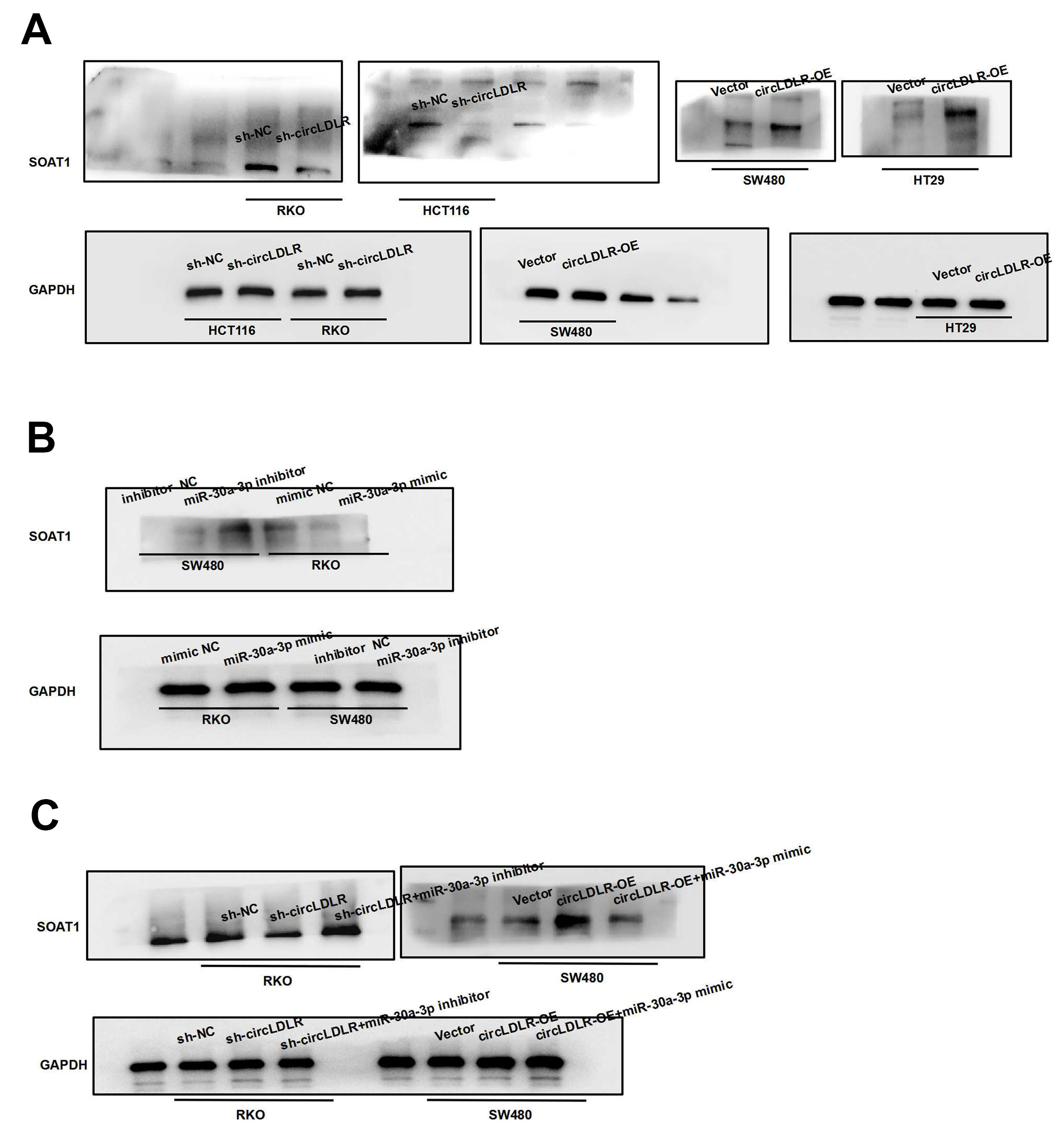

Supplement: Supplementary file 14 — Supplementary Fig. WB [file 41420_2022_1110_MOESM14_ESM.jpg]
