# Supplementary material for: Circular RNA circLDLR facilitates cancer progression by altering the miR-30a-3p/SOAT1 axis in colorectal cancer
Source: Cell Death Discov. 2022 Jul 11;8:314. doi: 10.1038/s41420-022-01110-5 (PMC9276972; doi:10.1038/s41420-022-01110-5)
Supplement: Supplementary file 15 — Recheck report [file 41420_2022_1110_MOESM15_ESM.pdf]

6.7-Wang

Circular RNA circLDLR facilitates cancer progression by altering the miR-30a-3p/SOAT1 axis in colorectal cancer

Ruoqin Wang[1,2,4], Jiayu Wang[1,2], Yanjun Chen[1,2], Yuqi Chen[1,2], Qinhua Xi[2], Linqing Sun[1,2], Xueguang Zhang[1,3,5], Guangbo Zhang[1,3,5], Xianglin Ding[6,*], Tongguo Shi[1,3,4,5,*], Weichang Chen[1,2,3,4,5,*]

[1]Jiangsu Institute of Clinical Immunology, The First Affiliated Hospital of Soochow University, 178 East Ganjiang Road, Suzhou, China.

[2]Department of Gastroenterology, The First Affiliated Hospital of Soochow University, 188 Shizi Road, Suzhou, China.

[3]Jiangsu Key Laboratory of Clinical Immunology, Soochow University, 178 East Ganjiang Road, Suzhou, China.

[4]Suzhou Key Laboratory for Tumor Immunology of Digestive Tract, The First Affiliated Hospital of Soochow University, 178 East Ganjiang Road, Suzhou, China.

[5]Jiangsu Key Laboratory of Gastrointestinal Tumor Immunology, The First Affiliated Hospital of Soochow University, 178 East Ganjiang Road, Suzhou, China.

[6]Department of Gastroenterology Suzhou Yongding Hospital, 1388 Gaoxin Road, Suzhou, China.

*Corresponding Author:

Weichang Chen, Mailing address: 50 Donghuan Road, Suzhou, Jiangsu Province, 215100, China; Phone: +86(512)67781318; Email: weichangchen@126.com

Tongguo Shi, Mailing address: 178 East Ganjiang Road, Suzhou, Jiangsu Province,215213, China.; Phone: +86(512)65125011; Email: shitg@suda.edu.cn

Xianglin Ding, Mailing address: 1388 Gaoxin Road, Suzhou, Jiangsu Province,215299, China.; Phone:+86(512)63180205; Email:Darnic5911@163.com

Abstract

Colorectal cancer (CRC) is the third most common malignancy worldwide. Circular RNAs (circRNAs) have been reported to play critical regulatory roles in tumorigenesis, serving as tumor biomarkers and therapeutic targets. However, the contributions of circRNAs to CRC tumorigenesis are unclear. In our study, high expression of circLDLR was found in CRC tissues and cells and was closely associated with the malignant progression and poor prognosis of CRC patients. We demonstrated that circLDLR boosts growth and metastasis of CRC cells in vitro and in vivo, and modulates cholesterol levels in vitro. Mechanistically, we showed that circLDLR competitively binds to miR-30a-3p and prevents it from reducing the SOAT1 level, facilitating the malignant progression of CRC. In sum, our findings illustrate that circLDLR participates in CRC tumorigenesis and metastasis via the miR-30a-3p/SOAT1 axis, serving as a potential biomarker and therapeutic target in CRC.

Keywords: colorectal cancer, circLDLR, miR-30a-3p, SOAT1

Introduction

Colorectal cancer (CRC) is one of the most common tumors and the leading cause of cancer-related death all over the world [1]. In spite of great improvements in therapeutic strategies, including colectomy, chemotherapy and immunotherapy, the high frequencies of recurrence and metastasis make CRC a serious menace to human health [2, 3]. CRC diagnosed at an advanced stage, particularly distant metastasis patients, remain a low 5-year survival rate [4, 5]. Thus, discovering new biomarkers for early diagnosis, precise metastasis prediction and prognosis is needed.

As a kind of abundant and ubiquitous noncoding RNAs, circular RNAs (circRNAs) have single-stranded closed-loop structures [6]. Accompanied by the continuous technical advance of the next-generation sequencing, circRNAs are potentiality for serving as tumor biomarkers and curative targets in the clinic [7]. Recently, literature have demonstrated that circRNAs partake in regulating miscellaneous tumor biological processes, such as invasion, metastasis, proliferation, tumor angiogenesis, drug resistance and cancer metabolism [8]. For example, circ-ERBIN is highly expressed and facilitates the proliferation, migration and metastasis in CRC [9]. Knockdown of circHIPK3 effectively inhibits various biological functions in CRC cells by sponging miR-7, such as proliferation, migration, and invasion [10]. Lei et al. noted that circCUL2 activated autophagy in a miR-142-3p/ROCK2 axis-dependent manner, and functioned as a tumor suppressor and regulator of resistance to cisplatin [11]. Moreover, circ-PVT1 activates miR-106a-5p/HK2 signaling, regulating biological processes, such as growth and metastasis, as well as glycolytic metabolism

in oral squamous cell carcinoma [12]. However, there is still no universally accepted explanation for how key differential circRNAs regulate CRC progression and metastasis.

In this work, we scouted the results of ribosomal RNA-depleted RNA-sequencing data for five CRC patients and focused on a circular RNA (circ_0006877) stemmed from the LDLR gene, labeled as circLDLR. And circLDLR was obviously boosted in both CRC tissues and CRC cell lines, and it was associated with a poor prognosis in CRC patients. Moreover, circLDLR was identified as a key regulator in CRC. In vitro and in vivo experiments displayed that it modulates CRC proliferation and metastasis. Our mechanistic study evidenced that circLDLR deeds as a blocker for miR-30a-3p to modulate the level of sterol O-acyltransferase 1 (SOAT1), further facilitating the tumorigenesis of CRC. Therefore, circLDLR has the potential to become promising therapeutic target for CRC.

Materials and methods

Human CRC tissue specimens

Five CRC tissue samples and paired adjacent normal tissue samples were obtained from patients who received surgery at the First Affiliated Hospital of Soochow University (Suzhou, China). We received approval from the Moral Principle Board of the First Affiliated Hospital of Soochow University before we collected the samples (2020084). Informed consent was signed by each CRC patient. Detailed clinicopathological characteristics of these patients are described in Supplementary Table S1.

Cell lines and cell cultures

The human HEK293T cells, normal colonic epithelial cell line NCM-460, and human CRC cell lines (HCT116, HT29, RKO, SW620, HCT8, LoVo, SW480, and Caco-2) were purchased from American Type Culture Collection (ATCC, USA). DMEM (Eallbio, Beijing, China), containing 10% fetal bovine serum (FBS, Eallbio) and 1% penicillin-streptomycin (Beyotime, Shanghai, China, #C0222), was used for all cells maintain in a humidified incubator with 5% CO_2 at 37°C.

Nucleic acid preparation and quantitative real-time polymerase chain reaction (qRT-PCR)

TRIzol reagent (Vazyme, Nanjing, China, #R401-01) was adopted to elicit total RNA from tissues or cultured cells. We extracted the nuclear and cytoplasmic fractions with Minute Cytoplasmic & Nuclear Extraction Kits for Cells (Invent Biotechnologies, Inc., Plymouth, MN, USA, #SC-003). Isolated RNA was used for reverse transcription with MonScript RT Super Mix with dsDNase (Monad, Wuhan, China, #MR05201) in the light of the manufacturer's manual. We performed the quantitative PCR with SYBR green using the ChemoHS qPCR Mix kit (Monad, Wuhan, China, #MQ00401) on a CFX96 Touch Real-Time-PCR system (Bio-Rad, CA, USA) in the light of the manufacturer's manual. We performed expression analysis with specific primers for each gene, which are shown in Table S2 in Supplementary materials.

RNase R treatment

We incubated total RNA from CRC cells with RNase R (Epicentre Technologies, USA). The condition of incubation with RNase R was at a concentration of 3u /mg at

37°C for 30 min in accordance with the manufacturer's protocol. We detected the stability of circLDLR and LDLR mRNA (mLDLR) using qRT-PCR.

Actinomycin D assay

RKO cells were forced on equally in 6-well plates (5×10^5 cells per well). We handled cells with 2 µg/ml actinomycin D (MCE, HY-17559) for several specific times (0 h, 4 h, 8 h, 12 h or 24 h). Then, we reaped the cells and used them to analyze the mRNA contents of the linear and circular forms of the LDLR gene using qRT-PCR. Then, we normalized the expression of mRNA to the values measured in the 0 h group.

Fluorescence in situ hybridization (FISH)

Cy3-labeled circLDLR probes (Geneseed, Guangzhou, China) and FAM-labeled miR-30a-3p probes (GenePharma, Shanghai, China) were used for the detection of the colocalization of circLDLR and miR-30a-3p in CRC cells. We marked cell nuclei using 4,6-diamidino-2-phenylindole (DAPI, #C1002). The Fluorescent In Situ Hybridization Kit (RiboBio, Guangzhou, China, #C10910) were utilized to discover the signals of the probes in the light of the manufacturer's instructions. Briefly, cells (1×10^5) were keeped in 15 mm cell culture dishes with glass-bottom. The next day, we used 4% paraformaldehyde to fix the cells at room temperature for 10 min, permeabilized the cells in Triton X-100 (0.5%) for 5 min at 4°C. Then we rinsed them in PBS. All the reagents were RNase-free. After incubating the cells for 30 min at 37°C in prehybridization buffer, we hybridized them with the probes in the hybridization buffer (1:50) overnight. Next, the cells were washed in different washing buffers in order, and the nuclei were stained with DAPI following the

manufacturer's protocol. We acquired all images on a confocal laser scanning microscope (Olympus FLUOVIEW FV1000).

Cell viability

To evaluate the proliferation of CRC cells, Cell Counting Kit-8 (CCK-8, NCM, Suzhou, China, #C6005) was applied. In the CCK-8 experiment, we cultured transfected CRC cells into a well of 96-well plate at a concentration of 5000 cells. At the same time every day, we added ten microliters of CCK-8 solution into each well , and the absorbance value was quantified at 450 nm as a reference.

5-Ethynyl-2′-deoxyuridine (EdU) incorporation assay

To perform the EdU experiment, we used the BeyoClick™ EdU Cell Proliferation Kit with Alexa Fluor 555 (Beyotime, #C0075S) in the light of the manufacturer's protocol. In Brief, we seeded cells (1×10^5) in 15 mm glass-bottom cell culture dishes. The next day, we incubated the cells with 10 µM EdU working solution and cultured them at 37°C with 5% CO_2 for 2 h. After fixed in paraformaldehyde (4%), the cells were permeabilized in 0.3% Triton X-100 and washed them in 3% BSA. Finally, we incubated the cells with Click Additive Solution and Hoechst 33342 in the light of the manufacturer's manual. Using a confocal laser scanning microscope (Olympus FLUOVIEW FV1000), we obtained all images. Finally, we calculated the percentage of EdU incorporation (DNA Synthesis) to evaluate cell proliferation.

Transwell migration and invasion assays

To carry out the migration assay, we resuspended cells (4×10^5 cells) in medium without FBS and added them to the upper chamber in a 24-well plate with the pore of

8 μm (BD Biosciences, NJ, USA, #353097). To perform the invasion assay, we coated the upper chambers with 100 μl of diluted Matrigel (200 μg/ml, Corning, Shanghai, China, #356234) for 2 h. Then we filled the bottom chamber with 800 μl of cell culture medium supplemented with 20% FBS as the attractant. After incubating the cells for 24 h, we fixed the cells with 4% paraformaldehyde for 15 min, which were remained on the bottom surface of the upper chamber. Then we stained these cells for 15 min with crystal violet (Beyotime, #C0121). Finally, we imaged the cells on the lower side of the chamber membrane and counted them under an inverted microscope.

Analysis of cellular cholesterol levels

The low-density lipoprotein cholesterol (LDL-C) and total cholesterol (T-CHO) kits (Nanjing Jiancheng Bioengineering Institution, Nanjing, China, #A111-1-1, #A113-1-1) were used to measure the concents of T-CHO and LDL-C in CRC cells.

Cell transfection and infection

Human CRC cell lines were cultured in a 6-well plate at 37°C in a humidified 5% CO_2 atmosphere overnight. CircLDLR-specific siRNA (siRNA-1, -2, -3, -4 and -5), miRNA mimics and miRNA inhibitors (GenePharma) were transfected with Lipofectamine 2000 (Invitrogen, USA) in the light of the manufacturer's protocol. Lentiviruses carrying a circLDLR overexpression vector or short hairpin RNA (shRNA) containing the sequence of circLDLR siRNA-2 was obtained from Geneseed (Guangzhou, China). We used an empty backbone vector as a control. When CRC cells grew to 30% confluence, lentiviral particles (MOI: 20) were used to infect them. We verified the effectiveness of overexpression or interference by

fluorescence microscopy and qRT-PCR after 72 h.

RNA Immunoprecipitation (RIP) assays

CRC cells (3×10^7) were cumulated and lysed using RIP lysis. The lysis buffer contained 2.5 mM $MgCl_2$, 60 U/ml Superase-In (Ambion, #AM2694), 20 mM Tris, 500 mM NaCl, 2% SDS, 1 mM DTT (Sigma, #43816), and protease inhibitors (Biotool, #B14001). Then, we subjected the lysates to sonication, and incubated the supernatants with an anti-AGO2 antibody (Proteintech, Wuhan, China, #10686-1-AP) or IgG overnight at 4°C. Then, we added Protein A/G beads (MedChemExpress, Monmouth Junction, NJ, USA, #HY-K0202) for incubation at 4°C for a further 3 h. After washing the protein with washing buffer (PBS, 0.5% Triton X-100, pH 7.4), we then purified the immunoprecipitated RNAs using TRIzol and assessed by qRT-PCR analysis.

RNA pull-down assay

Biotinylated circLDLR were designed and synthesized (GenePharma, Shanghai, China). We harvested approximately 3×10^7 circLDLR-overexpressing CRC cells, then lysed and sonicated them for further experiments. We incubated the biotinylated circLDLR probe with C-1 magnetic beads (Invitrogen) and cultured them at room temperature for 1 h, generating probe-coated beads. Then, we incubated cell lysates with the circLDLR probe at 4°C overnight, using oligo probe as control. After washing beads with wash buffer, we eluted and extracted the RNA transcripts bound to the beads with TRIzol for analysis.

Luciferase reporter assay

We seeded HEK293T cells (3×10^5) in 6-well plates, and cotransfected them with a mixture of miRNA mimics and luciferase reporter plasmid (1 μg) using Lipofectamine 2000 transfection reagent. 48 h later, we washed the cells with PBS and lysed them in Promega Passive Lysis Buffer. Then, we measured the luciferase activities by the Dual Luciferase Reporter Assay System (Promega, Madison, USA, #E1910) following the manufacturer's instructions. Finally, we normalized the luciferase values and calculated relative luciferase activity .

Western blot analysis

Our team drew proteins with RIPA lysis buffer (Beyotime, #P0013B) adding protease inhibitors (Beyotime, #P1045). Utilizing a BCA protein assay kit (Beyotime, #P0010), we determined the concentrations of proteins. Then, total proteins (30 μg) were separated by electrophoresis using the 10% ExpressCast PAGE Kit (NCM, Suzhou, China, #P2012) and transferred to PVDF membranes (GE Healthcare Life Science, Germany). We used 5% BSA (Fcmacs, Nanjing, China,#FMS-WB021) to block the membranes with for about 1 h, then incubated them with primary antibodies at 4°C overnight. We used the primary antibodies anti-GAPDH (Abclonal, Wuhan, China, #AC035) and anti-SOAT1 (CST, Beverly, Ma, USA, #35695S) in the light of the manufacturer's manuals. After the membranes marking using a secondary antibody for 1 h, we obtained images utilizing Imaging Systems of Bio-Rad ChemiDoc™ MP.

Animal experiments

In vivo experiments were authorized by the Institutional Animal Care and Use Committee of Soochow University (Suzhou, China; SUDA20210918A02). Animal

care and all experimental procedures were in the light of institutional ethical guidelines for animal experiments. Stable circLDLR-overexpressing or circLDLR-knockdown CRC cells and corresponding control cells were harvested and suspended in PBS. Each BALB/c nude mouse (six weeks old, female) was implanted subcutaneously with 5×10^6 cells in the right flank. The mice were monitored every other day to measure tumor weight and tumor volume. About 3 weeks after injection, we sacrificed the mice, then dissected and weighed the tumors. To bulid a metastasis model, 7-week-old BALB/c nude mice (female) were administered stable circLDLR-overexpressing or circLDLR-knockdown CRC cells (2×10^6 cells per mouse) via tail intravenous injection. After forty days, the lungs were surgically removed. Then, we fixed the lungs in 4% paraformaldehyde, then stained them with hematoxylin and eosin (HE). Lung metastatic foci were counted by two experienced pathologists.

Hematoxylin and eosin (HE) staining and immunohistochemistry (IHC) analysis

Paraffin sections (5-µm thick) from mouse tumor or lung tissues were used for HE and IHC analyses. HE staining was conducted following the manufacturer's instructions (Beyotime, #C0105). IHC analysis was conducted as described previously [13, 14]. Sections were processed and stained with an anti-Ki-67 antibody (BOSTER, California, USA, #BM4381, 1:50), anti-CD31 antibody (Abcam, Cambridge, MA, USA, #ab32457, 1:1500) or anti-SOAT1 antibody (CST, Beverly, Ma, USA, #35695S, 1:50).

Statistical analysis

GraphPad Prism version 6.0 (GraphPad Software, La Jolla, CA, USA) and SPSS version 26.0 (IBM Corp., Armonk, NY, USA) were adopted for statistical analysis. Overall survival comparisons were conducted by the log-rank (Mantel–Cox) test for Kaplan–Meier plots. The relationship between circLDLR and patient characteristics was detected using the chi-squared test. Data are presented as the mean ± standard deviation (SD). Intergroup differences were analyzed by Student's t test or one-way ANOVA. P values of < 0.05 were considered statistically significant.

Results

CircLDLR is upregulated in CRC tissues and positively associated with a poor prognosis in CRC patients.

We first performed RNA-seq analysis of ribosomal RNA-depleted total RNA from five clinic CRC tissue samples and the normal paired adjacent tissue samples. Then, we obtained the constructed circRNA profiling database, and we found that the detected circRNAs' length was mostly less than 1000 nucleotides (Fig. 1a). In total, differentially expressed 411 circRNAs ($P < 0.05$ and fold change > 2.0) were identified in the CRC tissues relative to the adjacent normal tissues (Fig. 1b). Among these circRNAs, 184 were significantly elevated, and 277 were lessed (Fig. 1b, c, Supplementary Fig. S1). Next, we put attention to the upregulated circRNA hsa_circ_0006877, which termed circLDLR in the remainder of the article and is assumed to be derived from the low-density lipoprotein receptor (LDLR) gene. Although circLDLR did not have the highest fold change value and P value among

the 184 upregulated circRNAs in the circRNA profiling, compared with other up-regulated circRNAs, the expression of circLDLR in 5 pairs of tissues has a better consistency. The expression of circLDLR was re-examined in 80 human CRC and 15 normal tissue specimens. As Fig. 1d displayed, the expression of circLDLR was obviously higher in CRC tissues than that in the adjacent tissues. Moreover, we observed that the circLDLR level of most CRC cell lines tested (HCT116, SW480, SW620, RKO, HCT8, HT29, LoVo and Caco-2) was higher than that of the colonic epithelial cell line NCM-460 (Fig. 1e).

Next, we investigated the relationships between clinicopathological characteristics and circLDLR expression in CRC patients. Correlation analysis manifested the expression of circLDLR was markedly related to the TNM stage of clinicopathological parameter (Supplementary Table S3). Besides, CRC patients who had higher circLDLR expression had poorer overall survival (OS) (Fig. 1g). Overall, our data show that circLDLR is abnormally expressed in CRC tissues and cell lines, has a positive relation to the poor prognosis of CRC patients.

Characterization of circLDLR

CircLDLR, a predicted length of 295 nt, arises from exons 13 and 14 of the LDLR gene and is located at chromosome 19p13.2 (Fig. 2a). Its precise genomic location is chr19:11,230,768-11,231,198 (GRCh38/hg38) (Fig. 2a). Subsequently, we used qRT-PCR to amplified the back-spliced junction of circLDLR with divergent primers, then verified them via Sanger sequencing (Fig. 2a). Furthermore, we detected circLDLR expression in cDNA and genomic DNA (gDNA) of RKO cells

using PCR with divergent primers or convergent primers and an agarose gel electrophoresis assay. Our consequences suggested that circLDLR was amplified from cDNA but not from gDNA by using divergent primers (Fig. 2b). A qRT-PCR assay with oligo (dT)18 primers showed that circLDLR had no poly-A tail (Fig. 2c). To investigate the stability of circLDLR, total RNA of RKO cells was treated with or without RNase R. As shown in Fig. 2d, circLDLR could resist digestion by RNase R, while linear LDLR mRNA (mLDLR) could be degraded by RNase R. Additionally, circLDLR showed a longer half-life than mLDLR in RKO cells after we added actinomycin D to them, which serves as an inhibitor of transcription (Fig. 2e). Subsequently, nuclear and cytoplasmic fractionation followed by qRT-PCR or FISH indicated that circLDLR was predominately positioned in the cytoplasm (Fig. 2f, g). In conclusion, circLDLR was substantiated to be a stable circular RNA that was principally positioned in the cytoplasm.

CircLDLR facilitates CRC malignant behaviour and increases cholesterol levels in vitro.

Trying to identify the specific function of circLDLR in our study, gain- and loss-of-function assays were executed. First, five short interfering RNAs (siRNAs) which were devised to target the back-splice site of circLDLR (si-LDLR-1, si-LDLR-2, si-LDLR-3, si-LDLR-4 and si-LDLR-5) were synthesized (Fig. 3a). Two of them (si-LDLR-2 and si-LDLR-4) specifically downregulated the expression of circLDLR in RKO and HCT116 cells without influencing the LDLR mRNA (mLDLR) level (Fig. 3a). To explore the influence of circLDLR on CRC cell proliferation,

CCK-8 and EdU assays were performed. We found knocking down circLDLR expression suppressed the rates of proliferation in CRC cells (Fig. 3b, c). The roles of circLDLR in modulating the CRC cell behaviour of migration and invasion was further exploded via Transwell assays. Our findings confirmed that the migration and invasion of CRC cells were significantly suppressed after transfection with si-LDLR-2 and si-LDLR-4 (Fig. 3d). Since LDLR is an key regulator of cholesterol homeostasis [15], we tried to discover whether circLDLR affects cholesterol metabolism of CRC cells. We observed that circLDLR knockdown markedly reduced the total cholesterol (T-CHO) and low-density lipoprotein cholesterol (LDL-C) expression levels of in CRC cells (Fig. 3e).

Furthermore, we ectopically expressed circLDLR to identify its biological function using a circLDLR-overexpression plasmid. As Fig. 4a shows, the content of circLDLR was significantly upregulated in SW480 and HT29 cells after transfection with circLDLR-overexpression plasmids. As expected, overexpression of circLDLR (circLDLR-OE) significantly facilitated the growth of SW480 and HT29 cells (Fig. 4b-c), which also boosted the migrative and invasive behaviour of these cells (Fig. 4d). Moreover, circLDLR overexpression led to the elevated contents of T-CHO and LDL-C in SW480 and HT29 cells (Fig. 4e). Overall, our consequents signified that circLDLR boosted cell proliferation and metastasis in CRC cells, and increased the contents of T-CHO and LDL-C.

CircLDLR deeds as a miRNA sponge for miR-30a-3p.

Given that circLDLR was predominantly positioned in the cytoplasm, which lacked

the ability to encode a protein (analyzed by circRNADb, http://202.195.183.4:8000/circrnadb/circRNADb.php) (Fig. 2f, g, Supplementary Fig.S2), we surmised that it might deed as a miRNA sponge. Therefore, an RIP using an anti-AGO2 antibody was performed. The findings suggested that circLDLR was specifically enriched in SW480 and HT29 cells by the AGO2-specific antibody after transfection with circLDLR-overexpression plasmids (Fig. 5a). Next, three public databases (miRanda, TargetScan and RNAhybrid) were combined to search for candidate target miRNAs of circLDLR. With these bioinformatic approaches, 109 candidate miRNAs potentially interacting with circLDLR were identified, as shown in Fig. 5b and Supplementary Table S4. Among these miRNAs, eleven potential target miRNAs, which were previously reported to be negatively associated with tumor progression, were selected for further analysis (Supplementary Table S5). Then, stable circLDLR-knockdown (sh-circLDLR) RKO and HCT116 cells and stable circLDLR-overexpressing SW480 and HT-29 cell lines were established (Supplementary Fig. S3) and exploited to disclose the expression of the eleven potential target miRNAs (Supplementary Fig. S4). As Fig. 5c shows, only the expression miR-30a-3p has the negative correlation with the circLDLR level in these four CRC cell lines, suggesting that circLDLR might interact with miR-30a-3p. Furthermore, the data of RNA pull-down assay evidenced that both circLDLR and miR-30a-3p were specifically enriched by the circLDLR probe compared with a negative control oligo probe in stable circLDLR-overexpressing SW480 and HT29 cells (Fig. 5d). We further confirmed that miR-30a-3p had a minimal modulatory role

in circLDLR production in CRC cells (Fig. 5e). Moreover, FISH analysis indicated miR-30a-3p colocalized with circLDLR in the CRC cell cytoplasm (Fig. 5f). To further address whether circLDLR functions by sponging miR-30a-3p, we performed a series of rescue experiments. An EdU assay indicated that circLDLR knockdown-induced suppressive effects on cell proliferation were reversed by treatment with miR-30a-3p inhibitors, while the promotive effects of circLDLR overexpression was abolished by treatment with miR-30a-3p mimics (Fig. 5g). In addition, the reduced cell migration and invasion of RKO arised from circLDLR knockdown was effectively abolished by knockdown of miR-30a-3p (Fig. 5h). Consistently, miR-30a-3p overexpression reversed the promotive consequences of circLDLR overexpression on the migrative and invasive behaviour of SW480 cells (Fig. 5h). Moreover, the introduction of a miR-30a-3p inhibitor abolished the suppressive influences of circLDLR deletion on the T-CHO and LDL-C expression (Fig. 5i). The decreased contents of T-CHO and LDL-C induced via circLDLR overexpression were reversed by treatment with miR-30a-3p mimics (Fig. 6i). Taken together, these data indicate that circLDLR facilitates CRC tumorigenesis and also increases the levels of T-CHO and LDL-C by sponging miR-30a-3p.

SOAT1 is a downstream target gene of miR-30a-3p.

CircLDLR is assumed to be derived from the low-density lipoprotein receptor (LDLR) gene, a key gene associated with cholesterol metabolism. Moreover, previous researches have indicated that cholesterol metabolism exerts crucial roles in cancer progression and metastasis [16, 17]. More importantly, we found that circLDLR not

only facilitates tumorigenesis in CRC, but also increases the levels of T-CHO and LDL-Cby sponging miR-30a-3p. Hence, we inferred that the circLDLR/miR-30a-3p axis might regulate cholesterol metabolism-related genes. According to TargetScan (http://www.targetscan.org/vert_72/), we screened out two key cholesterol metabolism-associated genes (SOAT1 and HMGCR) as the predicted downstream regulatory genes of miR-30a-3p. qRT-PCR results indicated that miR-30a-3p mimics could obviously decrease the expression of SOAT1, while miR-30a-3p inhibitors markedly increased the level of SOAT1 in both RKO cells and SW480 cells (Fig. 6a). However, the expression of miR-30a-3p had a smaller influence on the expression of HMGCR (Supplementary Fig. S5a, b). The models of hybridization between the SOAT1 3'UTR and miR-30a-3p are illustrated in Fig. S5c. A luciferase reporter assay showed that miR-30a-3p overexpression decreased the activity of the luciferase reporter containing the wild-type SOAT1 3'UTR (Fig. 6b). Moreover, overexpression of miR-30a-3p markedly decreased the SOAT1 protein level of RKO cells, and downregulation of miR-30a-3p produced the opposite results in SW480 cells (Fig. 6c). These results propound that SOAT1 is a direct target gene of miR-30-3p. Next, we explored whether circLDLR exerts a regulatory effect on SOAT1 expression via miR-30a-3p. Western blot data indicated that silencing of circLDLR obviously reduced SOAT1 expression in RKO and HCT116 cells, while overexpression of circLDLR increased that in SW480 and HT29 cells (Fig. 6d). Furthermore, the reduced SOAT1 expression caused by circLDLR knockdown was significantly inverted by treatment with miR-30a-3p inhibitors in RKO cells (Fig. 6e). Moreover,

overexpression of miR-30a-3p abolished the promotion of SOAT1 protein expression by circLDLR overexpression in SW480 cells (Fig. 6e). Collectively, these data propose that SOAT1 is a downstream target of circLDLR/miR-30a-3p.

The circLDLR/miR-30a-3p/SOAT1 axis modulates malignant behaviour and increases cholesterol levels in CRC.

To further explore the biological role of SOAT1, we examined whether SOAT1 participates in circLDLR-mediated CRC progression. EdU and Transwell assays were performed and revealed that knocking down SOAT1 inhibited cell growth, migration and invasion, which were enhanced in HT29 cells and SW480 cells after treatment with a miR-30a-3p inhibitor (Fig. 6f, g, Supplementary Fig. S6a, b). Moreover, SOAT1 downregulation significantly reversed the promotive effect of miR-30a-3p inhibitors on T-CHO and LDL-C contents in CRC cells (Fig. 6h). Consistently, knockdown of SOAT1 inverted the enhancement of proliferation, migration and invasion induced by circLDLR overexpression (Fig. 6i, j, Supplementary Fig. S6c, d), and reversed the promotive consequence of circLDLR overexpression on T-CHO and LDL-C contents in CRC cells (Fig. 6k). Our findings indicated that SOAT1 suppression could obviously restrain circLDLR-mediated tumorigenesis and the increase of cholesterol levels.

CircLDLR benefits tumor growth and metastasis of CRC in vivo

Our team further explored whether circLDLR contributes to CRC expansion in vivo, so we constructed xenograft mouse models. Stable circLDLR-knockdown HCT116 cells (sh-circLDLR) or stable circLDLR-overexpressing HT29 cells (circLDLR-OE)

were infused into the subcutaneous of right hind flank of nude mice. The results showed that circLDLR knockdown inhibited, while circLDLR overexpression promoted, tumor growth in vivo, as evidenced by tumor imaging and results for tumor volume and tumor weight (Fig. 7a-f). IHC staining showed that Ki-67, CD31 and SOAT1 levels were significantly positively correlative with circLDLR contents in xenograft tumor tissues (Supplementary Fig. S7). Conducting a tail vein injection model, we then analyzed the action of circLDLR on CRC metastasis in vivo. The findings pointed that circLDLR knockdown suppressed the metastasis of CRC cells to the lungs (Fig. 7g-i). CircLDLR overexpression had the opposite effect (Fig. 7j-l). Our findings suggest that circLDLR enhances CRC tumor expansion and metastasis in vivo.

Discussion

Currently, numerous circular RNAs have been identified and are considered tumor regulators in multiple cancers, including CRC [8]. For example, it has been reported that circPPP1R12A enhances the proliferation ability, as well as migration and invasive behaviours of colon cancer via Hippo-YAP signaling [18]. Silencing circDENND4C could block the miR-760/GLUT1 axis, which obviously suppresses cell expansion and migration, and glycolysis as well in CRC [19]. In our study, circRNA profiling of CRC tissues was performed by RNA-seq analysis of ribosomal RNA-depleted total RNA. Among the differential circRNAs, we focused on the upregulated circRNA circLDLR, which was positively related to the OS and TNM stage of CRC patients. Importantly, loss- and gain-of-function experiments signified

that circLDLR facilitated the abilities of proliferation, migration, and invasion in CRC cells in vitro and accelerated CRC tumorigenesis and metastasis in vivo. These results revealed that circLDLR is a novel oncogenic circRNA in CRC.

In this study, we further probed into the roles of circLDLR, which is derived from the LDLR gene, a key gene associated with cholesterol homeostasis. Cholesterol is an indispensable lipid component of various cell types and exerts critical effects on cell signaling. The cholesterol accumulation is a well-known feature of tumors, and picking out cholesterol metabolism has been explored as a novel therapeutic scheme for cancer treatment [20]. There are literatures that LDLR is the patriarch of the LDLR family, orchestrating cholesterol homeostasis [15]. Interestingly, exosomal circLDLR has been revealed to be associated with the production of estradiol in polycystic ovary syndrome [21]. We found that circLDLR increased the contents of T-CHO and LDL-C in CRC cells. However, we only detected the contents of T-CHO and LDL-C while did not elaborate on the mechanism of circLDLR in cholesterol metabolism in several parts of our study. We focused on the change of cholesterol levels in CRC cells and tried to provide an insight into the possibility of circLDLR in cholesterol metabolism. Collectively, our outcomes imply that circLDLR exerts a significant role in modulating cholesterol levels, which may be a key regulator of cholesterol metabolism in CRC.

CircRNAs have been demonstrated to play critical effects on CRC via various mechanisms, such as miRNA sponges [22]. As a sponge for miR-200c-3p, knockdown of Hsa_circ_001783 markedly inhibits the ability of proliferation and the behavior

invasion in breast cancer cells [23]. Circ-RanGAP1 sponges miR-877-3p to raise VEGFA production and boosts gastric cancer invasion and metastasis [24]. Herein, circLDLR was confirmed to be predominantly positioned in the cytoplasm of CRC cells, representing that circLDLR might deed as a miRNA sponge. Our results showed that among all the potential miRNAs bound by circLDLR, miR-30a-3p, which is downregulated in CRC tissues[25], interacted with circLDLR in CRC cells. Moreover, we demonstrated that circLDLR exerted its function as a ceRNA (competing endogenous RNA) through competitive binding to miR-30a-3p. It has been reported that circRNAs mediate their functions via other mechanisms, such as translation to produce functional peptides and interaction with RNA-binding proteins [22]. We used a detailed database named circRNADb, which contains human circRNAs with protein-coding annotations [26], to investigate whether circLDLR can be translated into a functional protein. Our results indicated circLDLR lacking a protein-coding sequence (Supplementary Fig. S2). However, we could not exclude the possibility that circLDLR exerts its function via other mechanisms.

SOAT1, also known as cholesterol acyltransferase 1 (ACAT1), is a key player in cellular cholesterol homeostasis [20]. It has been informed that SOAT1 is widely expressed in different types of cells, highly expressed in various tumors as well [27]. More importantly, nevanimibe HCl, a novel SOAT1 inhibitor, was used in a phase 1 study of adrenocortical carcinoma [28]. In the current study, our findings showed that the direct target gene of miR-30a-3p is SOAT1. CircLDLR could control SOAT1 production by restraining miR-30a-3p. Moreover, SOAT1 suppression could

significantly inhibit circLDLR-mediated tumorigenesis and the elevated contents of T-CHO and LDL-C in CRC cells. Therefore, the circLDLR/miR-30a-3p axis modulates CRC tumorigenesis and cholesterol levels via SOAT1.

In conclusion, we first demonstrated the clinical significance of circLDLR and revealed that circLDLR promoted CRC cell tumorigenesis and increased cholesterol levels via miR-30a-3p/SOAT1 signaling (Fig. 8). Based on our consequences, we trust that circLDLR may serve as a encouraging curative gene and prognostic predictor in CRC.

Abbreviations

CRC: Colorectal cancer; SOAT1: Sterol O-acyltransferase 1; CircRNAs: Circular RNAs; RIP: RNA immunoprecipitation; FISH: Fluorescence in situ hybridization; LDLR: Low-density lipoprotein receptor; OS: Overall survival; mLDLR: LDLR mRNA; CeRNA: Competing endogenous RNA.

Ethics approval and consent to participate

This study was approved by the First Affiliated Hospital of Soochow University (Suzhou, China; 2020084).

Consent for publication

Not applicable.

Competing interests

The authors declare that they have no competing interests.

Availability of data and materials

For all data requests, please contact the corresponding author.

Funding

This work was supported by the National Natural Science Foundation of China (82073156); Key Social Development Project of Jiangsu Province (BE2020656); The Natural Science Foundation of the Jiangsu Higher Education Institutions of China (20KJA310005); Suzhou Science & Technology Plan Project (SKJYD2021082); Project of Medical Research of Jiangsu Commission of Health (ZDA2020008); Suzhou Wujiang District Revitalizing Health through Science & Education Project (WWK202011).

Acknowledgements

Not applicable.

Authors' contributions

The authors checked and approved the final manuscript. XGZ, GBZ and TGS designed all experiments, YJC and YQC collected clinical samples, RQW and JYW performed all experiments. QHX and LQS performed the data analysis. The manuscript was wrote by RQW and revised by XLD, TGS and WCC.

References

1. Bray F, Ferlay J, Soerjomataram I, Siegel R, Torre L, and Jemal A. Global cancer statistics 2018: GLOBOCAN estimates of incidence and mortality worldwide for 36 cancers in 185 countries. CA: a cancer journal for clinicians 2018; 68:394-424.

2. Dienstmann R, Vermeulen L, Guinney J, Kopetz S, Tejpar S, and Tabernero J. Consensus molecular subtypes and the evolution of precision medicine in

colorectal cancer. Nature reviews. Cancer 2017; 17:79-92.

3. Watson A and Collins P. Colon cancer: a civilization disorder. Digestive diseases (Basel, Switzerland) 2011; 29:222-8.

4. Siegel R, Miller K, and Jemal A. Cancer statistics, 2016. CA: a cancer journal for clinicians 2016; 66:7-30.

5. Goldstein D, Zeichner S, Bartnik C, Neustadter E, and Flowers C. Metastatic Colorectal Cancer: A Systematic Review of the Value of Current Therapies. Clinical colorectal cancer 2016; 15:1-6.

6. Chen L. The biogenesis and emerging roles of circular RNAs. Nature reviews. Molecular cell biology 2016; 17:205-11.

7. Zhang Z, Yang T, and Xiao J. Circular RNAs: Promising Biomarkers for Human Diseases. EBioMedicine 2018; 34:267-274.

8. Beilerli A, Gareev I, Beylerli O, Yang G, Pavlov V, Aliev G, et al. Circular RNAs as biomarkers and therapeutic targets in cancer. Seminars in cancer biology 2021.

9. Chen L, Wang L, Ren Y, Pang Z, Liu Y, Sun X, et al. The circular RNA circ-ERBIN promotes growth and metastasis of colorectal cancer by miR-125a-5p and miR-138-5p/4EBP-1 mediated cap-independent HIF-1α translation. Molecular cancer 2020; 19:164.

10. Zeng K, Chen X, Xu M, Liu X, Hu X, Xu T, et al. CircHIPK3 promotes colorectal cancer growth and metastasis by sponging miR-7. Cell death & disease 2018; 9:417.

11. Peng L, Sang H, Wei S, Li Y, Jin D, Zhu X, et al. circCUL2 regulates gastric cancer malignant transformation and cisplatin resistance by modulating autophagy activation via miR-142-3p/ROCK2. Molecular cancer 2020; 19:156.

12. Zhu X, Du J, and Gu Z. Circ-PVT1/miR-106a-5p/HK2 axis regulates cell growth, metastasis and glycolytic metabolism of oral squamous cell carcinoma. Molecular and cellular biochemistry 2020; 474:147-158.

13. Wang R, Ma Y, Zhan S, Zhang G, Cao L, Zhang X, et al. B7-H3 promotes colorectal cancer angiogenesis through activating the NF-κB pathway to induce VEGFA expression. Cell death & disease 2020; 11:55.

14. Shi T, Ma Y, Cao L, Zhan S, Xu Y, Fu F, et al. B7-H3 promotes aerobic glycolysis and chemoresistance in colorectal cancer cells by regulating HK2. Cell death & disease 2019; 10:308.

15. Go G and Mani A. Low-density lipoprotein receptor (LDLR) family orchestrates cholesterol homeostasis. The Yale journal of biology and medicine 2012; 85:19-28.

16. Zhao Z, Hao D, Wang L, Li J, Meng Y, Li P, et al. CtBP promotes metastasis of breast cancer through repressing cholesterol and activating TGF-β signaling. Oncogene 2019; 38:2076-2091.

17. Wang C, Li P, Xuan J, Zhu C, Liu J, Shan L, et al. Cholesterol Enhances Colorectal Cancer Progression via ROS Elevation and MAPK Signaling Pathway Activation. Cellular physiology and biochemistry : international

journal of experimental cellular physiology, biochemistry, and pharmacology 2017; 42:729-742.

18. Zheng X, Chen L, Zhou Y, Wang Q, Zheng Z, Xu B, et al. A novel protein encoded by a circular RNA circPPP1R12A promotes tumor pathogenesis and metastasis of colon cancer via Hippo-YAP signaling. Molecular cancer 2019; 18:47.

19. Zhang Z, Zhang Y, Qin X, Wang Y, and Fu J. Circular RNA circDENND4C facilitates proliferation, migration and glycolysis of colorectal cancer cells through miR-760/GLUT1 axis. European review for medical and pharmacological sciences 2020; 24:2387-2400.

20. Xu H, Zhou S, Tang Q, Xia H, and Bi F. Cholesterol metabolism: New functions and therapeutic approaches in cancer. Biochimica et biophysica acta. Reviews on cancer 2020; 1874:188394.

21. Huang X, Wu B, Chen M, Hong L, Kong P, Wei Z, et al. Depletion of exosomal circLDLR in follicle fluid derepresses miR-1294 function and inhibits estradiol production via CYP19A1 in polycystic ovary syndrome. Aging 2020; 12:15414-15435.

22. Long F, Lin Z, Li L, Ma M, Lu Z, Jing L, et al. Comprehensive landscape and future perspectives of circular RNAs in colorectal cancer. Molecular cancer 2021; 20:26.

23. Liu Z, Zhou Y, Liang G, Ling Y, Tan W, Tan L, et al. Circular RNA hsa_circ_001783 regulates breast cancer progression via sponging

miR-200c-3p. Cell death & disease 2019; 10:55.

24. Lu J, Wang Y, Yoon C, Huang X, Xu Y, Xie J, et al. Circular RNA circ-RanGAP1 regulates VEGFA expression by targeting miR-877-3p to facilitate gastric cancer invasion and metastasis. Cancer letters 2020; 471:38-48.

25. Ma Y, Zhang P, Yang J, Liu Z, Yang Z, and Qin H. Candidate microRNA biomarkers in human colorectal cancer: systematic review profiling studies and experimental validation. International journal of cancer 2012; 130:2077-87.

26. Chen X, Han P, Zhou T, Guo X, Song X, and Li Y. circRNADb: A comprehensive database for human circular RNAs with protein-coding annotations. Scientific reports 2016; 6:34985.

27. Wang Y, Bian Y, Luo J, Lu M, Xiong Y, Guo S, et al. Cholesterol and fatty acids regulate cysteine ubiquitylation of ACAT2 through competitive oxidation. Nature cell biology 2017; 19:808-819.

28. Smith D, Kroiss M, Kebebew E, Habra M, Chugh R, Schneider B, et al. A phase 1 study of nevanimibe HCl, a novel adrenal-specific sterol O-acyltransferase 1 (SOAT1) inhibitor, in adrenocortical carcinoma. Investigational new drugs 2020; 38:1421-1429.

Figure legends

Fig. 1 CircLDLR is elevated in CRC tissues and positively related with a poor prognosis in patients with CRC. a The length distribution of exonic circRNAs. **b, c** Schematic illustration of the differentially expressed circRNAs in CRC tissues. **d** The fold change in circLDLR expression between CRC tissues and normal adjacent tissues was analyzed by qRT-PCR. **e** Relative expression of circLDLR in CRC cell lines compared to that in the NCM-460 cell line. **f** Kaplan-Meier analysis of the overall survival of CRC patients stratified into high and low circLDLR expression groups. *$P < 0.05$; ***$P < 0.001$; NS, no significance.

Fig. 2 circLDLR characterization in colorectal cancer. a Schematic diagram of circLDLR shows that it is generated by the circlization of LDLR exons 13 and 14. CircLDLR was scouted by qRT-PCR, which sequence was confirmed via Sanger sequencing. Red arrows used to indicate the special splicing junction of circLDLR. **b** CircLDLR expression in RKO cells detected by qRT-PCR followed by agarose gel electrophoresis showing that divergent primers amplified circLDLR in cDNA but not genomic DNA (gDNA). GAPDH served as a negative control. **c** After reverse transcription using random hexamer or oligo (dT)18 primers, the relative expression of circLDLR and mLDLR was analyzed by qRT-PCR. **d** Relative RNA contents were detected by qRT-PCR, then normalized to the value analyzed in the mock group. **E** Relative expression of circLDLR and mLDLR was detected by qRT-PCR after we treated them using actinomycin D at the several time points. **f** The abundance of circLDLR and mLDLR in cytoplasmic and nuclear parts of RKO cells was evaluated

by qRT-PCR. We applied U6 and GAPDH as positive controls for the cytoplasmic and nuclear fractions, respectively. **g** The localization of circLDLR in RKO cells was scouted by FISH. Nuclei were stained with DAPI (blue), and circLDLR probes were labeled with Cy3 (red). Scale bar, 20 µm. CircLDLR, the circular RNA derived from exons 13 and 14 of the LDLR gene; Data are presented as the mean±SD of three independent experiments. $^{**}P < 0.01$; $^{***}P < 0.001$.

Fig. 3 CircLDLR suppression inhibits CRC malignant behaviour and increases cholesterol levels in vitro. a The circLDLR and mLDLR expression was assessed in RKO and HCT116 cells transfected with five independent siRNAs targeting circLDLR by qRT-PCR. **b** Cell proliferation was scouted at the indicated time points by CCK-8 assays evaluating HCT116 cells and RKO cells stimulated with si-NC or si-circLDLR. **c** EdU analysis of the proliferative ability of HCT116 cells and RKO cells treated with si-NC or si-circLDLR. Representative images are shown. Scale bar, 200 µm. Statistical analysis of the EdU-positive cell percentage in transfected GC cells is shown in the bar graph. **d** The cell migration and invasion of CRC cells were examined by Transwell assays after cells were transfected with si-circLDLR or si-NC. Representative images are shown. Scale bar, 200 µm. Statistical analysis of the migrated and invaded cell numbers is shown in the bar graph. **e** The production of T-CHO and LDL-C was assessed in CRC cells transfected with si-NC or si-circLDLR. Data are presented as the mean±SD of three independent experiments. $^{*}P < 0.05$; $^{**}P < 0.01$; $^{***}P < 0.001$.

Fig. 4 CircLDLR overexpression facilitates CRC malignant behaviour and

increases cholesterol levels in vitro. **a** The expression of circLDLR in RKO and HCT116 cells transfected with a circLDLR-overexpression vector (circLDLR-OE) or the control vector (Vector) was assessed by qRT-PCR. **b** Cell proliferation was scouted at the indicated time points by CCK-8 assays evaluating RKO cells and HCT116 cells transfected with circLDLR-OE or Vector. **c** EdU analysis of the proliferative ability of RKO cells and HCT116 cells transfected with circLDLR-OE or Vector. Representative images are shown. Scale bar, 200 µm. Statistical analysis of the EdU-positive cell percentage in transfected CRC cells is shown in the bar graph. **d** The cell migration and invasion of CRC cells were examined by Transwell assays after cells were transfected with circLDLR-OE or Vector. Representative images are shown. Scale bar, 200 µm. Statistical analysis of the migrated and invaded cell numbers is shown in the bar graph. **e** The production of T-CHO and LDL-C in CRC cells transfected with circLDLR-OE or Vector were appraised. Data are presented as the mean±SD of three independent experiments. $*P < 0.05$; $**P < 0.01$; $***P < 0.001$.

Fig. 5 CircLDLR acts as a miRNA sponge for miR-30a-3p. **a** RIP assays were carried out using an AGO2-specific antibody and SW480 or HT29 cells, and the richness of circLDLR was scouted by qRT-PCR. **b** Schematic illustration showing the 109 overlapping miRNAs with potential binding to circLDLR predicted by miRanda, RNAhybrid and TargetScan. **c** Schematic illustration showing that miR-30a-3p was verified to be one of the 11 predicted miRNAs associated with tumor progression among the 109 overlapping miRNAs in different CRC cell lines. **d** Lysates prepared from circLDLR-OE SW480 and HT29 cells were incubated with biotinylated probes

against circLDLR, and then an RNA pull-down assay was performed. qRT-PCR was carried out to prove the contents of circLDLR and miR-30a-3p. **e** The production of circLDLR in different CRC cell lines transfected with miR-30a-3p mimics or mimic NC were evaluated by qRT-PCR. **f** The circLDLR and miR-30a-3p colocalization in RKO cells was detected by RNA FISH. Nuclei were stained with DAPI (blue). CircLDLR probes were labeled with Cy3 (red), and miR-30a-3p probes were labeled with FAM (green). Scale bar, 20 µm. **g** EdU analysis of the proliferative ability of RKO cells transfected with sh-circLDLR or cotransfected with sh-circLDLR and miR-30a-3p inhibitors. EdU analysis of the proliferative ability of SW480 cells treated with circLDLR-OE or co-transfected with circLDLR-OE and miR-30a-3p mimics. Representative images are shown. Scale bar, 200 µm. Statistical analysis of the EdU-positive cell percentage in transfected RKO and SW480 cells is displayed in the bar graph. **h** The cell migration and invasion of RKO cells transfected with sh-circLDLR or cotransfected with sh-circLDLR and miR-30a-3p inhibitors were examined. The cell migration and invasion of SW480 cells transfected with circLDLR-OE or co-stimulated with circLDLR-OE and miR-30a-3p mimics were examined. Representative images are shown. Scale bar, 200 µm. Statistical analysis of the migrated and invaded cell numbers is shown in the bar graph. **i** The T-CHO and LDL-C contents in RKO cells transfected with sh-circLDLR or cotransfected with sh-circLDLR and miR-30a-3p inhibitors were assessed. The T-CHO and LDL-C contents in SW480 cells treated with circLDLR-OE or cotransfected with circLDLR-OE and miR-30a-3p mimics were assessed. Data are presented as the

mean±SD of three independent experiments. $^{**}P < 0.01$; $^{***}P < 0.001$; NS, no significance.

Fig. 6 SOAT1 is a downstream target of miR-30a-3p. a RKO and SW480 cells were transfected with miR-30a-3p mimics or inhibitors. SOAT1 expression was scouted by qRT-PCR. **b** Relative activities of luciferase were measured in HEK293T cells after transfection with SOAT1-Mut or SOAT1-WT and miR-30a-3p mimics or mimic NC. **c** Overexpression of miR-30a-3p restrained the protein production of SOAT1. Suppression of miR-30a-3p raised the expression of SOAT1 protein. **d** Knockdown of circLDLR (sh-circLDLR) inhibited the protein expression of SOAT1. Overexpression of circLDLR (circLDLR-OE) increased the protein expression of SOAT1. **e** Knockdown of miR-30a-3p reversed the sh-circLDLR-induced downregulation of SOAT1 expression in RKO cells. Overexpression of miR-30a-3p reversed the circLDLR-OE-induced upregulation of SOAT1 expression in SW480 cells. GAPDH deeded as a loading control. **f** EdU analysis of the cell proliferation ability in SW480 and HT29 transfected with miR-30a-3p inhibitors or cotransfected with si-SOAT1 and miR-30a-3p inhibitors. **g** Cell migration and invasion in SW480 and HT29 transfected with miR-30a-3p inhibitors or cotransfected with si-SOAT1 and miR-30a-3p inhibitors were examined. **h** The contents of T-CHO and LDL-C in SW480 and HT29 cells transfected with miR-30a-3p inhibitors or cotransfected with si-SOAT1 and miR-30a-3p inhibitors were assessed. **i** EdU analysis of the cell proliferation ability in SW480 and HT29 transfected with circLDLR-OE or cotransfected with si-SOAT1 and circLDLR-OE. **j** Cell behaviors of migration and

invasion in HT29 and SW480 cells transfected with circLDLR-OE or cotransfected with si-SOAT1 and circLDLR-OE were examined. **k** The contents of T-CHO and LDL-C in SW480 and HT29 cells transfected with circLDLR-OE or cotransfected with si-SOAT1 and circLDLR-OE were assessed. Data are presented as the mean±SD of three independent experiments. *P < 0.05; **P < 0.01; ***P < 0.001; NS, no significance.

Fig. 7 CircLDLR knockdown restrains CRC tumor growth and metastasis in vivo. a Representative images of subcutaneous xenograft tumors from sh-circLDLR group and the sh-NC group (n=5). **b**, **c** In comparison with the sh-NC group, the sh-circLDLR group had tumors with observably decreased volume (**b**) and weight (**c**). **d** Representative images of subcutaneous xenograft tumors from the circLDLR-OE group and the Vector group (n=5). **e**, **f** In comparison with the Vector group, the circLDLR-OE group had tumors with observably increased volume (**e**) and weight (**f**). **g** Representative images of mouse lungs six weeks after transplantation. **h, i** Images of HE staining are shown (**h**), and the tumor area was measured (**i**). **j** Representative images of mouse lungs six weeks after transplantation. **k, l** Images of HE staining are shown (**k**), and the tumor area was measured (**l**). Data are presented as the mean±SD of three independent experiments. *P < 0.05; **P < 0.01; ***P < 0.001.

Fig. 8 Schematic diagram showing the regulatory mechanisms of the circLDLR/miR-30a-3p/SOAT1 axis in CRC.

6.7-Wang

原创性报告

29%	23%	26%	3%
相似指数	网际网络来源	出版物	学生文稿

主要来源

1	molecular-cancer.biomedcentral.com<br>网际网络来源	6%
2	www.nature.com<br>网际网络来源	3%
3	www.ncbi.nlm.nih.gov<br>网际网络来源	2%
4	pubmed.ncbi.nlm.nih.gov<br>网际网络来源	1%
5	Youxia Wang, Xiaoling Chen, Zhiqing Huang, Daiwen Chen, Bing Yu, Jie Yu, Hong Chen, Jun He, Yuheng Luo, Ping Zheng. "Dietary Ferulic Acid Supplementation Improves Antioxidant Capacity and Lipid Metabolism in Weaned Piglets", Nutrients, 2020<br>出版物	1%
6	www.frontiersin.org<br>网际网络来源	1%
7	Yizheng Wu, Ziang Xie, Junxin Chen, Jiaxin Chen et al. "Circular RNA circTADA2A promotes osteosarcoma progression and	1%

metastasis by sponging miR-203a-3p and regulating CREB3 expression", Molecular Cancer, 2019
出版物

8	test.dovepress.com<br>网际网络来源	1 %
9	www.jcancer.org<br>网际网络来源	<1 %
10	Chengdong Liu, Xiaohan Zhou, Qian Long, Hanyi Zeng, Qingcan Sun, Yuting Chen, Dehua Wu, Li Liu. "Small extracellular vesicles containing miR-30a-3p attenuate the migration and invasion of hepatocellular carcinoma by targeting SNAP23 gene", Oncogene, 2020<br>出版物	<1 %
11	Jian Yu, Qing-guo Xu, Zhen-guang Wang, Yuan Yang, Ling Zhang, Jin-zhao Ma, Shu-han Sun, Fu Yang, Wei-ping Zhou. "Circular RNA cSMARCA5 inhibits growth and metastasis in hepatocellular carcinoma", Journal of Hepatology, 2018<br>出版物	<1 %
12	Xiaomi Li, Huimin Lu, Yanzheng Gu, Xueguang Zhang, Guangbo Zhang, Tongguo Shi, Weichang Chen. "Tim-3 suppresses the killing effect of Vγ9Vδ2 T cells on colon cancer cells by reducing perforin and granzyme B	<1 %

expression", Experimental Cell Research, 2020
出版物

13	assets.researchsquare.com<br>网际网络来源	<1%
14	Qi Xu, Demin Cheng, Guanru Li, Yi Liu, Ping Li, Wenqing Sun, Dongyu Ma, Chunhui Ni. "CircHIPK3 regulates pulmonary fibrosis by facilitating glycolysis in miR-30a-3p/FOXK2-dependent manner", International Journal of Biological Sciences, 2021<br>出版物	<1%
15	breast-cancer-research.biomedcentral.com<br>网际网络来源	<1%
16	onlinelibrary.wiley.com<br>网际网络来源	<1%
17	"17th International Congress of Immunology, 19–23 October 2019, Beijing, China", European Journal of Immunology, 2019<br>出版物	<1%
18	Jingbing Lou, Hongliang Zhang, Jiuhui Xu, Tingting Ren, Yi Huang, Xiaodong Tang, Wei Guo. "circUSP34 accelerates osteosarcoma malignant progression by sponging miR_16_5p", Cancer Science, 2021<br>出版物	<1%
19	Ruoqin Wang, Linqing Sun, Suhua Xia, Hongya Wu et al. "B7-H3 suppresses doxorubicin-	<1%

induced senescence-like growth arrest in colorectal cancer through the AKT/TM4SF1/SIRT1 pathway", Cell Death & Disease, 2021
出版物

20	Xin Huang, Jingyu Wang, Jibin Guan, Zhong Zheng, JunFeng Hao, Zitong Sheng, Menghua Wang, Tianhua Xu, Guangying Guo, Li Yao. "Exosomal Circsafb2 Reshaping Tumor Environment to Promote Renal Cell Carcinoma Progression by Mediating M2 Macrophage Polarization", Frontiers in Oncology, 2022<br>出版物	<1 %
21	link.springer.com<br>网际网络来源	<1 %
22	Xiaobo Li, Binliang Wang, Mao Huang, Xiaomi Wang. "miR-30a-3p participates in the development of asthma by targeting CCR3", Open Medicine, 2020<br>出版物	<1 %
23	Taoyue Yang, Peng Shen, Qun Chen, Pengfei Wu et al. "FUS-induced circRHOBTB3 facilitates cell proliferation via miR-600/NACC1 mediated autophagy response in pancreatic ductal adenocarcinoma", Journal of Experimental & Clinical Cancer Research, 2021<br>出版物	<1 %

24	Yang Ji, Shikun Yang, Xueqi Yan, Li Zhu et al. "CircCRIM1 Promotes Hepatocellular Carcinoma Proliferation and Angiogenesis by Sponging miR-378a-3p and Regulating SKP2 Expression", Frontiers in Cell and Developmental Biology, 2021<br>出版物	<1 %
25	Www.dovepress.com<br>网际网络来源	<1 %
26	bio-bigdata.hrbmu.edu.cn<br>网际网络来源	<1 %
27	www.dovepress.com<br>网际网络来源	<1 %
28	Rongrong Huang, Yuan Zhang, Bing Han, Ying Bai, Rongbin Zhou, Guangming Gan, Jie Chao, Gang Hu, Honghong Yao. " Circular RNA regulates astrocyte activation via cooperation of autophagy and ER stress by targeting ", Autophagy, 2017<br>出版物	<1 %
29	jcancer.org<br>网际网络来源	<1 %
30	www.spandidos-publications.com<br>网际网络来源	<1 %
31	Defeng Li, Meifeng Yang, Aijun Liao, Bing Zeng et al. "Linc00483 as ceRNA regulates	<1 %

proliferation and apoptosis through activating MAPKs in gastric cancer", Journal of Cellular and Molecular Medicine, 2018
出版物

32	Xiaomin Li, Jianjun Wang, Chao Zhang, Chun Lin, Jianming Zhang, Wei Zhang, Wenjuan Zhang, Yanxia Lu, Lin Zheng, Xuenong Li. " Circular RNA circITGA7 inhibits colorectal cancer growth and metastasis by modulating the Ras pathway and upregulating transcription of its host gene ", The Journal of Pathology, 2018<br>出版物	<1 %
33	academic.oup.com<br>网际网络来源	<1 %
34	Jiacheng Cao, Xing Zhang, Penghui Xu, Haixiao Wang et al. "Circular RNA circLMO7 acts as a microRNA-30a-3p sponge to promote gastric cancer progression via the WNT2/β-catenin pathway", Journal of Experimental & Clinical Cancer Research, 2021<br>出版物	<1 %
35	aging-us.com<br>网际网络来源	<1 %
36	Penghui Xu, Xing Zhang, Jiacheng Cao, Jing Yang et al. "The Therapeutic Effect of Circular RNA ST3GAL6 on Blocking GC Malignant Behaviors Through Autophagy Regulated by	<1 %

the FOXP2/MET/Mtor Axis", Research Square Platform LLC, 2021
出版物

37 Yuan, Yunfeng, Yaxing Shen, Liang Xue, and Hong Fan. "miR-140 Suppresses Tumor Growth and Metastasis of Non-Small Cell Lung Cancer by Targeting Insulin-Like Growth Factor 1 Receptor", PLoS ONE, 2013.
出版物
<1%

38 Qiuli Wang, Dong Liang, Peng Shen, Yang Yu, Yuan Yan, Wei You. "Hsa_circ_0092276 promotes doxorubicin resistance in breast cancer cells by regulating autophagy via miR-348/ATG7 axis", Translational Oncology, 2021
出版物
<1%

39 Sen-Miao Tong, Ding-Yi Wang, Qing Cai, Sheng-Hua Ying, Ming-Guang Feng. "Opposite Nuclear Dynamics of Two FRH-Dominated Frequency Proteins Orchestrate Non-Rhythmic Conidiation in Beauveria bassiana", Cells, 2020
出版物
<1%

40 Wei Liu, Weiming Ma, Yuan Yuan, Youwei Zhang, Sanyuan Sun. "Circular RNA hsa_circRNA_103809 promotes lung cancer progression via facilitating ZNF121-dependent MYC expression by sequestering miR-4302",
<1%

Biochemical and Biophysical Research Communications, 2018
出版物

41	www.tandfonline.com<br>网际网络来源	<1 %
42	Feige Nian, Jing Zhu, Haining Chang. "Long non-coding RNA ANGPTL1-3 promotes multiple myeloma bortezomib resistance by sponging miR-30a-3p to activate c-Maf expression", Biochemical and Biophysical Research Communications, 2019<br>出版物	<1 %
43	Jensen, Gasim, Ismail, Gaafar, Kurtzhals, Kemp, El Hassan, Kharazmi, Theander. " Humoral and Cellular Immune Responses to Synthetic Peptides of the Kinetoplastid Membrane Protein_11 ", Scandinavian Journal of Immunology, 2008<br>出版物	<1 %
44	Tingting An, Tianxiu Dong, Haoxin Zhou, Yaodong Chen, Jiuwei Zhang, Yu Zhang, Zizhuo Li, Xiuhua Yang. "The transcription factor Krüppel-like factor 5 promotes cell growth and metastasis via activating PI3K/AKT/Snail signaling in hepatocellular carcinoma", Biochemical and Biophysical Research Communications, 2019<br>出版物	<1 %

45 Www.ijbs.com
网际网络来源 <1%

46 Jing Lin, Shasha Liao, E. Li, Zewa Liu, Ruihua Zheng, Xiaohua Wu, Wanting Zeng. "circCYFIP2 Acts as a Sponge of miR-1205 and Affects the Expression of Its Target Gene E2F1 to Regulate Gastric Cancer Metastasis", Molecular Therapy - Nucleic Acids, 2020
出版物 <1%

47 Dandan Li, Li Shen, Mengjie Xu, Xudong Zhang, Zhen Chen, Pan Huang, Congcong Huang, Shanshan Qin. "The SNAI2-ELF3-AS1 Feedback Loop Drives Gastric Cancer Metastasis and Regulates ELF3 Expression At Transcriptional and Post-Transcriptional Levels", Research Square Platform LLC, 2021
出版物 <1%

48 Huanji Xu, Sheng Zhou, Qiulin Tang, Hongwei Xia, Feng Bi. "Cholesterol metabolism: New functions and therapeutic approaches in cancer", Biochimica et Biophysica Acta (BBA) - Reviews on Cancer, 2020
出版物 <1%

49 Richard Greil, Evelyn Hutterer, Tanja Nicole Hartmann, Lisa Pleyer. "Reactivation of dormant anti-tumor immunity – a clinical perspective of therapeutic immune <1%

checkpoint modulation", Cell Communication and Signaling, 2017
出版物

50	Tingting Wang, Zhenchuan Liu, Yunlang She, Jiajun Deng et al. "A novel protein encoded by circASK1 ameliorates gefitinib resistance in lung adenocarcinoma by competitively activating ASK1-dependent apoptosis", Cancer Letters, 2021<br>出版物	<1 %
51	Yanjun Chen, Jiayu Wang, Juntao Li, Jinghan Zhu, Ruoqin Wang, Qinhua Xi, Hongya Wu, Tongguo Shi, Weichang Chen. "Astragalus polysaccharide prevents ferroptosis in a murine model of experimental colitis and human Caco-2 cells via inhibiting NRF2/HO-1 pathway", European Journal of Pharmacology, 2021<br>出版物	<1 %
52	jneuroinflammation.biomedcentral.com<br>网际网络来源	<1 %
53	journals.lww.com<br>网际网络来源	<1 %
54	www.researchsquare.com<br>网际网络来源	<1 %
55	Chen Lu, Dawei Rong, Bingqing Hui, Xuezhi He, Wei Jiang, Yetao Xu, Hongyong Cao,	<1 %

Zekuan Xu, Weiwei Tang. "CircETFA upregulates CCL5 by sponging miR-612 and recruiting EIF4A3 to promote hepatocellular carcinoma", Cell Death Discovery, 2021
出版物

---

56 Hong Mai, Yan Liao, Sufang Luo, Kaiyi Wei, Feng Yang, Haijuan Shi. "Histone deacetylase HDAC2 silencing prevents endometriosis by activating the HNF4A/ARID1A axis", Journal of Cellular and Molecular Medicine, 2021
出版物
<1 %

---

57 James R Hill, Xia Shao, Jay S. Wright, Jenelle Stauff et al. " Synthesis and evaluation of C- and F-labeled SOAT1 inhibitors as macrophage foam cell imaging agents ", ACS Medicinal Chemistry Letters, 2020
出版物
<1 %

---

58 Kaixuan Zeng, Xiaoxiang Chen, Mu Xu, Xiangxiang Liu, Xiuxiu Hu, Tao Xu, Huiling Sun, Yuqin Pan, Bangshun He, Shukui Wang. "CircHIPK3 promotes colorectal cancer growth and metastasis by sponging miR-7", Cell Death & Disease, 2018
出版物
<1 %

---

59 Lei Xing, Rui Yang, Xiaosong Wang, Xiaying Zheng, Xin Yang, Luyu Zhang, Rong Jiang, Guosheng Ren, Junxia Chen. "The circRNA circIFI30 promotes progression of triple-
<1 %

negative breast cancer and correlates with prognosis", Aging, 2020
出版物

60	Xiaoxiang Chen, Kaixuan Zeng, Mu Xu, Xiuxiu Hu, Xiangxiang Liu, Tao Xu, Bangshun He, Yuqin Pan, Huiling Sun, Shukui Wang. "SP1-induced lncRNA-ZFAS1 contributes to colorectal cancer progression via the miR-150-5p/VEGFA axis", Cell Death & Disease, 2018<br>出版物	<1 %
61	coek.info<br>网际网络来源	<1 %
62	jeccr.biomedcentral.com<br>网际网络来源	<1 %
63	stemcellres.biomedcentral.com<br>网际网络来源	<1 %
64	www.medicinalgenomics.com<br>网际网络来源	<1 %
65	Fagen Li, Ke Cao, Maoyun Wang, Yi Liu, Yin Zhang. "Astragaloside IV exhibits anti-tumor function in gastric cancer via targeting circRNA dihydrolipoamide S-succinyltransferase (circDLST)/miR-489-3p/eukaryotic translation initiation factor 4A1(EIF4A1) pathway", Bioengineered, 2022<br>出版物	<1 %

lr.vanderbilt.edu

66	网际网络来源	<1 %
67	Linyuan Xue, Hongyu Qi, He Zhang, Lu Ding, Qingxia Huang, Daqing Zhao, Boyang Jason Wu, Xiangyan Li. "Targeting SREBP-2-Regulated Mevalonate Metabolism for Cancer Therapy", Frontiers in Oncology, 2020<br>出版物	<1 %
68	Peng-Fei Zhang, Yi-Fan Xu, Ling-Xian Zhang, Jing Wu, Yue Fan. "CircRUNX1 Drives Immune Evasion and anti-PD1 Immunotherapy Resistance in Lung Adenocarcinoma by the miR-4739/PCSK9/MHC I Axis", Research Square Platform LLC, 2021<br>出版物	<1 %
69	Tomoko Kurata, Violeta Lukic, Miki Kozuki, Daisuke Wada et al. "Long-term Effect of Telmisartan on Alzheimer's Amyloid Genesis in SHR-SR After tMCAO", Translational Stroke Research, 2014<br>出版物	<1 %
70	Xiaoling Cai, Xueying Gao, Wenjia Yang, Yifei Chen, Simin Zhang, Lingli Zhou, Xueyao Han, Linong Ji. "No disparity of the efficacy and all cause mortality between Asian and non-Asian type 2 diabetes patients with SGLT2 inhibitors treatment: A meta-analysis", Journal of Diabetes Investigation, 2017	<1 %

出版物

71 Yanqing Feng, Gang Wei, Linfei Zhang, Huadong Zhou, Wei Wang, Peng Guo, Caitao Cheng, Lei Ji, Qinghe Cai, Yong Feng, Huahua Tu. "LncRNA DARS-AS1 aggravates the growth and metastasis of hepatocellular carcinoma via regulating the miR-3200-5p-Cytoskeleton associated protein 2 (CKAP2) axis", Bioengineered, 2021 <1 %
出版物

72 Yaxin Guo, Yuying Guo, Chen Chen, Dandan Fan, Xiaoke Wu, Luyang Zhao, Bo Shao, Zhenqiang Sun, Zhenyu Ji. "Circ3823 contributes to growth, metastasis and angiogenesis of colorectal cancer: involvement of miR-30c-5p/TCF7 axis", Molecular Cancer, 2021 <1 %
出版物

73 patents.google.com <1 %
网际网络来源

74 thno.org <1 %
网际网络来源

75 www.hindawi.com <1 %
网际网络来源

76 Lin Li, Jinhai Gou, Tao Yi, Zhengyu Li. "MicroRNA-30a-3p regulates epithelial-mesenchymal transition to affect embryo <1 %

implantation by targeting Snai2†", Biology of Reproduction, 2019
出版物

77 Ruoqin Wang, Yanchao Ma, Shenghua Zhan, Guangbo Zhang, Lei Cao, Xueguang Zhang, Tongguo Shi, Weichang Chen. "B7-H3 promotes colorectal cancer angiogenesis through activating the NF-κB pathway to induce VEGFA expression", Cell Death & Disease, 2020 <1 %
出版物

78 Zeyou Guo, Luyang Zhao, Suhui Ji, Ting Long, Yanling Huang, Rui Ju, Wei Tang, Weidong Tian, Jie Long. "CircRNA-23525 regulates osteogenic differentiation of adipose-derived mesenchymal stem cells via miR-30a-3p", Cell and Tissue Research, 2020 <1 %
出版物

79 Zihao Pan, Jianye Cai, Jiatong Lin, Huinian Zhou et al. "A novel protein encoded by circFNDC3B inhibits tumor progression and EMT through regulating Snail in colon cancer", Molecular Cancer, 2020 <1 %
出版物

80 A. Kolyada, Q. Ke, I. Karageorgos, P. Mahlawat, D. A. Barrios, P. M. Kang, N. Beglova. "Soluble analog of ApoER2 targeting beta2-glycoprotein I in immune complexes <1 %

counteracts hypertension in lupus-prone mice with spontaneous antiphospholipid syndrome", Journal of Thrombosis and Haemostasis, 2016
出版物

81 Hanchen Xu, Yujing Liu, Peiqiu Cheng, Chunyan Wang, Yang Liu, Wenjun Zhou, Yangxian Xu, Guang Ji. "CircRNA_0000392 promotes colorectal cancer progression through the miR-193a-5p/PIK3R3/AKT axis", Journal of Experimental & Clinical Cancer Research, 2020
出版物
<1 %

82 Hanqing Hong, Hai Zhu, Shujun Zhao, Kaili Wang et al. "The novel circCLK3/miR-320a/FoxM1 axis promotes cervical cancer progression", Cell Death & Disease, 2019
出版物
<1 %

83 Liang Chen, Ge Shan. "CircRNA in cancer: Fundamental mechanism and clinical potential", Cancer Letters, 2021
出版物
<1 %

84 Martínez-Oliván, Juan, Hugo Fraga, Xabier Arias-Moreno, Salvador Ventura, and Javier Sancho. "Intradomain Confinement of Disulfides in the Folding of Two Consecutive Modules of the LDL Receptor", PLoS ONE, 2015.
出版物
<1 %

85	Qiuling Niu, Zhijie Dong, Min Liang, Yuanwei Luo, Hai Lin, Mingzhen Lin, Xiu Zhong, Wenxia Yao, Jinsheng Weng, Xinke Zhou. "Circular RNA hsa_circ_0001829 promotes gastric cancer progression through miR-155-5p/SMAD2 axis", Journal of Experimental & Clinical Cancer Research, 2020<br>出版物	<1 %
86	Ting-Chen Chang. "Effect of conjugated equine estrogen in combination with two different progestogens on the risk factors of coronary heart disease in postmenopausal Chinese women in Taiwan: a randomized one-year study", Acta Obstetricia Et Gynecologica Scandinavica, 7/2004<br>出版物	<1 %
87	Yanchao Ma, Shenghua Zhan, Huimin Lu, Ruoqin Wang et al. "B7-H3 regulates KIF15-activated ERK1/2 pathway and contributes to radioresistance in colorectal cancer", Cell Death & Disease, 2020<br>出版物	<1 %
88	Yoshitoshi Ichikawa, Hidekazu Takahashi, Yoshinao Chinen, Asami Arita et al. "Low G9a expression is a tumor progression factor of colorectal cancer via IL-8 promotion", Carcinogenesis, 2022<br>出版物	<1 %

89 Yuanfang Li, Pengtao Yuan, Shengxin Fan, Bin Zhai et al. "miR-30a-3p can inhibit the proliferation and promote the differentiation of chicken primary myoblasts", British Poultry Science, 2022
出版物

<1 %

---

90 Zhenqiang Sun, Chunlin Ou, Weiguo Ren, Xiang Xie, Xiayu Li, Guiyuan Li. "Downregulation of long non-coding RNA ANRIL suppresses lymphangiogenesis and lymphatic metastasis in colorectal cancer", Oncotarget, 2016
出版物

<1 %

不含引文	关闭	不含相符结果	关闭
排除参考书目	开		
