# Supplementary material for: Circular RNA circLDLR facilitates cancer progression by altering the miR-30a-3p/SOAT1 axis in colorectal cancer
Source: Cell Death Discov. 2022 Jul 11;8:314. doi: 10.1038/s41420-022-01110-5 (PMC9276972; doi:10.1038/s41420-022-01110-5)
Supplement: Supplementary file 16 — DECLARATION OF CONTRIBUTIONS TO ARTICLE [file 41420_2022_1110_MOESM16_ESM.pdf]

DECLARATION OF CONTRIBUTIONS TO ARTICLE

ADMC

Manuscript Number: CDDISCOVERY-22-3918R1

Journal Name: **Cell Death Discovery** (the 'Journal')

Proposed Title of the Contribution: Circular RNA circLDLR facilitates cancer progression by altering the miR-30a-3p/SOAT1 axis in colorectal cancer (the 'Contribution)

Author(s): Ruoqin Wang, Jiayu Wang, Yanjun Chen, Yuqi Chen, Qinhua Xi, Linqing Sun, Xueguang Zhang, Guangbo Zhang, Xianglin Ding, Tongguo Shi, Weichang Chen (the 'Authors')

For all *Cell Death Discovery* articles, each person named as an author in the published version must be able to show he or she has contributed substantially to the article.

Authorship credit should be based on 1) substantial contributions to conception and design, acquisition of data, or analysis and interpretation of data; 2) drafting the article or revising it critically for important intellectual content; and 3) final approval of the version to be published. Authors should meet conditions 1, 2 and 3.

Any person who cannot be shown to have made a substantial contribution to the article cannot be listed as an author in the final version. The name of any person who is deemed to have made a minor contribution can, however, appear in the Acknowledgments section of the article.

Please complete the table below to indicate the contributions of all named authors to the manuscript.

Author Full Name:	Specification of Contribution to the Manuscript:
Ruoqin Wang	acquisition of data, drafting the article
Jiayu Wang	acquisition of data
Yanjun Chen	collected clinical samples
Yuqi Chen	collected clinical samples
Qinhua Xi	analysis and interpretation of data
Linqing Sun	analysis and interpretation of data
Xueguang Zhang	conception and design,revising it critically for important intellectual content
Guangbo Zhang	conception and design, final approval of the version to be published
Xianglin Ding	conception and design,revising it critically for important intellectual content
Tongguo Shi	conception and design,revising it critically for important intellectual content
Weichang Chen	conception and design,revising it critically for important intellectual content

Please complete the table below to indicate the contributions of all named authors to the figures.

Figure 1:

Ruoqin Wang, Jiayu Wang, Yanjun Chen,Yuqi Chen, Xianglin Ding, Tongguo Shi, Weichang Chen

Figure 2:

Ruoqin Wang, Jiayu Wang, Yanjun Chen,Yuqi Chen, Xianglin Ding, Tongguo Shi, Weichang Chen

Figure 3-5:

Ruoqin Wang, Jiayu Wang, Qinhua Xi, Linqing Sun, Guangbo Zhang, Xueguang Zhang, Xianglin Ding, Tongguo Shi, Weichang Chen

Figure 6:

Ruoqin Wang,Qinhua Xi, Linqing Sun, Guangbo Zhang, Weichang Chen

Figure 7:

Ruoqin Wang, Yanjun Chen,Yuqi Chen, Guangbo Zhang, Weichang Chen

Figure 8:

Ruoqin Wang, Jiayu Wang,Tongguo Shi, Weichang Chen

Signed for and on behalf of the Author(s):	Print Name:	Date:
Tongguo Shi	Tongguo Shi	June 24, 2022
